# Treatment with anticancer drugs for advanced pancreatic cancer: a systematic review

**DOI:** 10.1186/s12885-023-11207-4

**Published:** 2023-08-12

**Authors:** Josefina Salazar, Javier Bracchiglione, Olga Savall-Esteve, Alba Antequera, David Bottaro-Parra, Marta Gutiérrez-Valencia, Susana Martínez-Peralta, Carles Pericay, Ariadna Tibau, Xavier Bonfill, Appropriateness of Systemic Oncological Treatments for Advanced Cancer (ASTAC) Research Group, Roberto Acosta-Dighero, Ariadna Auladell-Rispau, Yahveth Cantero-Fortiz, Edgar Hernandez, Juan Irassar, Adriana-G Meade, Pamela Meinardi, Angela Merchán-Galvis, Nicolas Meza, María Jesús Quintana, Carolina Requeijo, Gerardo Rodriguez-Grijalva, Karla Salas-Gama, Marilina Santero, Olga Savall-Esteve, Anna Selva, Ivan Solà, Gerard Urrútia

**Affiliations:** 1 Iberoamerican Cochrane Centre, Biomedical Research Institute Sant Pau (IIB Sant Pau), Barcelona, Spain; 2 CIBER Epidemiología Y Salud Pública (CIBERESP), Barcelona, Spain; 3 Interdisciplinary Centre for Health Studies CIESAL, Universidad de Valparaíso, Viña del Mar, Chile; 4 International Health Department, ISGlobal, Hospital Clínic - Universitat de Barcelona, Barcelona, Spain; 5 Unitat de Cures Pal·Liatives de L’Institut d’Oncologia de La Catalunya Sud, Hospital Universitari Sant Joan de Reus, Tarragona, Spain; 6 Unit of Innovation and Organization, Navarre Health Service, Pamplona, Spain; 7 Navarre Institute for Health Research (IdiSNA), Pamplona, Spain; 8 Unitat Cures Pal·Liatives, Hospital de La Santa Creu I Sant Pau, Barcelona, Spain; 9 Servicio de Oncología Médica, Fundació Assistencial Mûtua Terrassa, Terrassa – Barcelona, Spain; 10 Oncology Department, Hospital de La Santa Creu I Sant Pau, Barcelona, Spain; 11 Institut d’Investigació Biomèdica Sant Pau, Barcelona, Spain; 12 Universitat Autònoma Barcelona, Barcelona, Spain

**Keywords:** Pancreatic Neoplasms, Antineoplastic agents, Immunotherapy, Systematic Review, Palliative care

## Abstract

**Background:**

Patients with advanced pancreatic cancer have a poor prognosis and high burden of cancer-related symptoms. It is necessary to assess the trade-off of clinical benefits and possible harms of treatments with anticancer drugs (TAD). This systematic review aims to compare the effectiveness of TAD versus supportive care or no treatment, considering all patient-important outcomes.

**Methods:**

We searched PubMed, Embase, Cochrane Library, and Epistemonikos. Two reviewers performed selection, data extraction and risk of bias assessment. We assessed certainty of the evidence using the GRADE approach.

**Results:**

We included 14 randomised controlled trials. Chemotherapy may result in a slight increase in overall survival (MD: 2.97 months (95%CI 1.23, 4.70)) and fewer hospital days (MD: -6.7 (-8.3, -5.1)), however, the evidence is very uncertain about its effect on symptoms, quality of life, functional status, and adverse events. Targeted/biological therapy may result in little to no difference in overall survival and a slight increment in progression-free survival (HR: 0.83 (95%CI 0.63, 1.10)), but probably results in more adverse events (RR: 5.54 (95%CI 1.24, 23.97)). The evidence is very uncertain about the effect of immunotherapy in overall survival and functional status.

**Conclusions:**

The evidence is very uncertain about whether the benefits of using treatment with anticancer drugs outweigh their risks for patients with advanced pancreatic cancer. This uncertainty is further highlighted when considering immunotherapy or a second line of chemotherapy and thus, best supportive care would be an appropriate alternative. Future studies should assess their impact on all patient-important outcomes to inform patients in setting their goals of care.

**Supplementary Information:**

The online version contains supplementary material available at 10.1186/s12885-023-11207-4.

## Background

Pancreatic cancer (PC) has the highest incidence-to-mortality ratio of any solid tumour, accounting for almost as many deaths as new cases in 2020 [1]. Most patients are diagnosed at an incurable, advanced stage, either regional (28%) or distant (48%) [2, 3]. The overall prognosis of these patients is very poor, with 1- and 5-year survival rates of 59% and 14% for regional stage, and of 21% and 3% for distant stage at diagnosis [2].

PC patients present a high burden of cancer-related symptoms, including pain, weight loss, fatigue, depression, and anxiety, which tend to increase closer to the end-of-life (EoL) period [4, 5]. Therefore, for patients with advanced PC and a poor prognosis, it seems coherent that treatment goals should mostly prioritise the improvement on quality of life [6].

The most widely used approach for advanced PC is treatment with anticancer drugs (TAD). Currently, guidelines recommend chemotherapy as first line therapy for locally advanced and metastatic PC in patients with an Eastern Cooperative Oncology Group (ECOG) performance status (PS) 0, 1 or 2 [7–9]. However, these treatments are usually suggested on the basis of a few weeks to months survival advantage, but do not explicitly assess and report the impact these treatments may have in terms of improving symptom burden, toxicity, functional status, and quality of life near death. More recently, other TADs are being evaluated, mainly immunotherapy, —due to promising results in other malignancies— [10, 11] and targeted/biological therapies —due to the advances in molecular characterization of PC [12].

However, through previous stages of our broad evidence synthesis project [13] we found that previous systematic reviews (SRs) that compared TAD with supportive care (SC) or no treatment in patients with advanced PC did not find conclusive evidence to support one option over the other. Moreover, we found that there were relevant primary studies that were not included in those SRs [14]. Therefore, we think it is still reasonable to compare TADs with SC or no treatment to assess the trade-off of clinical benefits and possible harms and in consequence, we aimed to conduct a new SR, following strict methodological guidelines, to assess the efficacy of TAD versus SC or no treatment in people with advanced PC, considering all patient-important outcomes.

## Methods

We conducted a SR adhering the Preferred Reporting Items for Systematic Reviews and Meta-Analyses (PRISMA) checklist [15]. The protocol for this study was prospectively registered and is publicly available in Open Science Framework [16].

This study is the last part of a three-stage comprehensive evidence synthesis project (ASTAC project) [16]. Briefly, we first conducted an overview of SRs [13] and then a scoping review and evidence map of primary and secondary studies assessing the effectiveness of TAD versus SC/no treatment for advanced PC [14].

### Eligibility criteria

We used the PICO framework to determine our research question and guide our eligibility criteria [17]. The clinical question was: *Are TADs (chemotherapy, immunotherapy or targeted/biological therapies) more effective compared to SC/no treatment in patients with advanced PC?*

#### Types of studies

We included randomised clinical trials (RCTs) and excluded quasi-experimental studies, observational studies, and reviews. For studies with more than two arms, we included those of interest only if the authors provided the necessary disaggregated data.

#### Types of participants

We considered studies including adults with primary or recurrent advanced PC. We considered PC as advanced when in stage III or IV, or when considered as such by the study authors. Authors may refer to this as incurable, secondary, metastatic, terminal, or progressive cancer. We excluded neuroendocrine neoplasms.

#### Types of interventions

For the experimental arm, we considered any TAD (chemotherapy, immunotherapy, or targeted/biological therapies), either monotherapy or in combination, whether individual or combined, with or without SC. We excluded trials that evaluated surgery or radiotherapy without TADs, intraperitoneal chemotherapy, and studies that consider chemotherapy only as adjuvant/neoadjuvant therapy or maintenance therapy.

For the control arm, we considered any supportive treatment, administered with the purpose of symptomatic or palliative control. This comprehends either usual treatment, SC, or best supportive care (BSC) [18, 19]. We also included trials that did not explicitly define the intervention, or using with placebo. We excluded studies if the control arm included any type of TAD or treatments with non-palliative intention (e.g., radiotherapy or surgery with curative intention).

#### Types of outcomes

We considered studies that reported any of the following outcomes.

1. Primary outcomes: overall survival (OS), quality of life (QoL), progression-free survival (PFS), functional status (FS), and toxicity measured as moderate or severe adverse events
2. Secondary outcomes: symptoms related to the disease, admissions to hospital or long-term centre, or emergency consultations, and quality of death (QoD) measured as admissions to hospital at the EoL (last 30 days of life), palliative care provided during the last year and/or place of death.

### Search methods for identification of studies

We identified potentially eligible references through a search strategy conducted for the first two stages of the broad evidence synthesis project [16], which involved a literature search in MEDLINE (access via PubMed), Embase (access via Ovid), the Cochrane Database of Systematic Reviews, CENTRAL, and Epistemonikos from inception onwards. We updated this search on April 2022, using the same search strings for each electronic database, but only focused on PC (Additional file 1). We also searched in clinicaltrials.gov to identify protocols of potentially eligible studies. In addition to electronic database searches, we asked experts in the field for relevant studies. We conducted a forward and backward citation search starting from the included studies, using citationchaser software [20–22]. We did not apply language restrictions.

### Selection of studies

Two reviewers independently screened the results of the search, first by title and abstract and in a second stage by full text. A third reviewer solved any disagreement in both stages. For this process, we used Rayyan, a web-based software platform [23].

### Data extraction and risk of bias assessment

Two reviewers independently extracted data using a sheet that was previously piloted. We solved disagreements through discussion. We extracted the following characteristics of the included RCTs: year of publication, country, number of arms, study phase, number of randomised and analysed participants, characteristics of included participants, of interventions and comparisons, outcomes assessed, results, conflicts of interest, and funding.

Two reviewers independently assessed the risk of bias at outcome level using the Cochrane Risk of Bias 2 (RoB2) tool [24]. We solved disagreements by consensus or a third reviewer.

### Data synthesis and analysis

We calculated mean difference (MD) for continuous outcomes and risk ratios (RR) for dichotomous outcomes, with their respective 95% confidence interval. When the number of events was zero in a treatment arm, we followed Cochrane Handbook guidance [25]. For survival outcomes we also used hazard ratios (HR) extracted from the published data [26].

When a study included multiple arms, we only considered comparisons relevant for our review, and when two comparisons (e.g., drug A versus placebo and drug B versus placebo) were combined in the same study, we followed the guidance of the Cochrane Handbook for Systematic Reviews of Interventions, to avoid double-counting [27].

### Data synthesis

We performed a meta-analysis using a random effects model for outcomes where studies were reasonably homogeneous (both clinically and methodologically), using ReviewManager (RevMan) 5.4. For other cases, we report results descriptively.

For each intervention, we grouped studies and considered all possible interventions in the control group (i.e., placebo, SC, BSC, usual treatments and others) as one unique comparator. We also performed subgroup analyses according to the lines of therapy [28].

We were not able to conduct the other pre-planned subgroup analysis [16] due to lack of information. Due to the small number of studies included in the meta-analyses, we were unable to analyse publication bias through funnel plots.

### Assessment of heterogeneity

We assessed heterogeneity both visually and using I^2^ through Software Review Manager 5.4. Cut-off values for I^2^ are not absolutes. An I^2^ below 40% might not be important and between 50%-90% may represent substantial heterogeneity. We considered the heterogeneity assessment for purposes of the estimation of certainty of evidence.

### Assessment of certainty of evidence

We assessed the certainty of the evidence according to GRADE guidance [29] and made a Summary of Findings (SoF) table for all outcomes. We classified the certainty of the evidence for each outcome as high, moderate, low, or very low. We initially rated the certainty for each outcome as high, since data comes from RCT, and we lowered in presence of important bias, indirectness, inconsistency, imprecision, or suspicion of publication bias.

## Results

### Description of studies

#### Results of the search

Through the previous stages of our broad evidence synthesis project [14], we identified 43 studies, with 59 references, that included participants with advanced PC and were included in our scoping review and evidence map. We identified 337 additional references through our search update. After removing duplicates, we screened 286 references by title and abstract, and finally, 6 full-text articles for inclusion. We identified one reference corresponding to the protocol registration of an RCT already included in the previous stages of our project. Therefore, we included a total of 14 RCTs, with 21 references, to answer our review question.

**Fig. 1:**
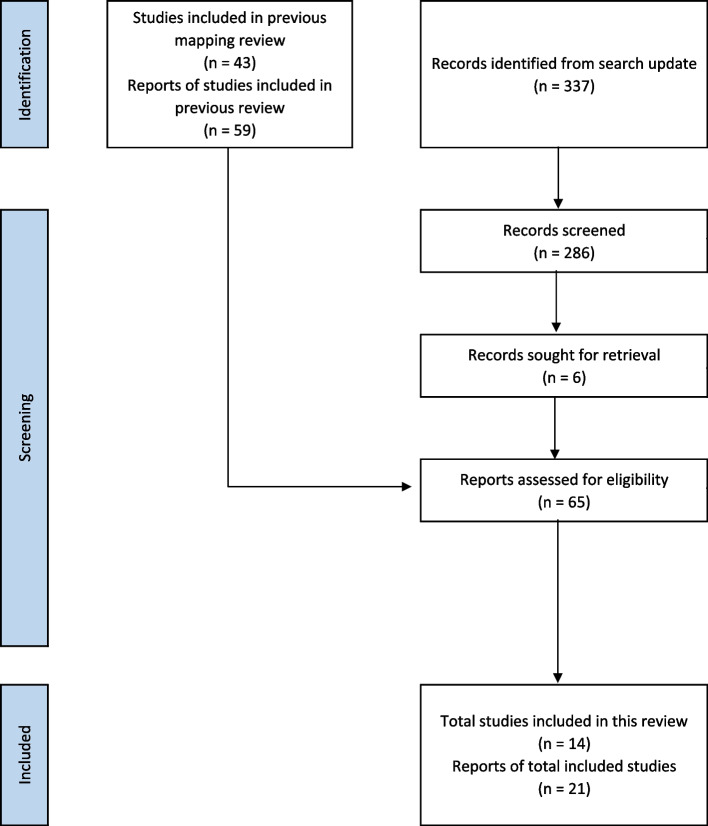
PRISMA flow chart: summary of the selection process

See Fig. 1 for the PRISMA flow chart. See additional file 2 for the reasons for exclusion of 44 reports.

#### Included Studies

See Table 1 for characteristics of the included studies and Table 2 for baseline characteristics of the included participants. See additional file 3 for the studies’ patient eligibility criteria and intervention details.

**Table 1:** Characteristics of included studies

Study ID	Country	Study design—Setting	Funding	Conflicts of interest	Participants	Intervention		Comparison
						Description	Line of therapy	
Chemotherapy								
Frey, 1981 [30]	US	Phase not specified) – Multicentre	Not specified/Not clear	Not declared	Histologically confirmed unresectable PC	**5-FU + CCNU + Celiotomy /Biliary bypass**	First line	**Palliative Surgery**
Glimelius, 1996 [31]	Sweden	Not specified	Private	Not declared	Histologically confirmed non curable PAC KPS ≥ 50%	**5-FU + Leucovorin ± Etoposide + BSC**	First line	**BSC**
Huguier, 2001 [32]	France	Phase III– Multicentre	Not specified/Not clear	Not declared	Histologically confirmed unresectable PAC	**5-FU + Cisplatin + Leucovorin + SC**	First line	**SC**
Mallinson, 1980 [33] & ABS [34]	UK	Phase not specified) – Multicentre	Public	Not declared	Adults from 35 to 75 years Unresectable PC	**5-FU + Methrotexate + Vincristine + Cyclophosphamide**	First line	**SC**
The Gastrointestinal Tumor Study Group, 1979 [35]	USA	Phase not specified) – Multicentre	Not specified/Not clear	Not declared	Histologically confirmed unresectable locally advanced PAC	Intervention 1: **5-FU + 4000R** Intervention 2: **5-FU + 6000R**	First line	**Palliative RT**
Xinopoulos, 2008 [36] & [37]	Greece	Phase not specified) – Multicentre	Not specified/Not clear	Not declared	Adults from 25 to 75 years Histologically or cytologically confirmed locally advanced PC KPS > 50%	**Gemcitabine + Palliative surgery**	First line	**Palliative Surgery**
Ciuleanu, 2009 [38]	Romania	Phase III – Multicentre	Private	Declared: Employees/consultants and stock holdings in funding company	≥ 18 years Metastatic PAC Progressed during or after treatment with gemcitabine	**Glufosfamide + BSC**	Second line	**BSC**
Pelzer, 2011 [39] ABS [40] TR: ISRCTN52780546	Germany	Phase III – Multicentre	Public and Private	Declared: None	≥ 18 years Histologically confirmed advanced PAC Progressed during first line gemcitabine	**OFF (5-FU + Oxaliplatin + Leucovorin)**	Second line	**BSC**
Palmer, 1994 [41]	UK	Phase not specified) – Single centre	Not specified/Not clear	Not declared	Unresectable advanced PC	**5-FU + Adriamycin + Mitomycin**	Not specified	**SC**
Shinchi, 2002 [42]	Japan	Phase not specified) – Single centre	Not specified/Not clear	Not declared	Histologically or cytologically confirmed unresectable locally advanced PC – KPS ≥ 60%	**5-FU + EBRT**	Not specified	**BSC**
Takada, 1998 [43]	Japan	Phase not specified) – Multicentre	Not specified/Not clear	Not declared	< 75 years Histologically confirmed unresectable PC	**Modified FAM (5-FU + Doxorubicin + Mitomycin) + Palliative surgery**	Not Specified	**Palliative Surgery**
Immunotherapy								
Oortgiesen, 2010 [44]	USA	Not specified	Not specified/Not clear	Declared: Financial interest in the product of the intervention	Adult patients Histologically or cytologically confirmed unresectable advanced PC	**PAS Vaccination**	Not Specified	**Placebo**
Gilliam, 2012 [45] & ABS [46]	UK, Hungary, Russia	Phase III – Multicentre	Private	Declared: None	Histologically confirmed advanced PC unsuitable or unwilling to receive CT	**G17DT: Antigastrin immunogen**	First line	**Placebo**
Targeted/biological therapy								
Propper, 2014 [47] & ABS [48] TR: NCT00674973	England	Phase II – Multicentre	Private	Declared: Employees/consultants and with stock holdings in the pharmaceutical company that financed the study	≥ 18 years Histologically or cytologically confirmed unresectable locally advanced or metastatic PC Failed on prior CT or were deemed unsuitable for first-line chemotherapy	**Erlotinib**	Not specified	**Placebo**

*RCT* Randomized clinical trial, *PC* Pancreatic cancer; *PAC* Pancreatic adenocarcinoma, *PS* Performace status, *KPS*, Karnosfky PS, *CT* chemotherapy, *RT* radiotherapy, *BSC* Best supportive care, *SC* Supportive care, *CCNU* Lomustine, 1—[2-chlorethyl—3-cyclohexyl—1 nitrosourea, *5FU* Fluorouracil, *EBRT* External beam radiotherapy, *PAS* Polyclonal antibody stimulator, *NR* Not reported, *ABS* Abstract, *TR* Trial registry

**Table 2:** Baseline characteristics of included participants

Study ID	N total Intervention vs Comparison	Age	% Female Intervention vs Comparison	Race/ethnicity %	Baseline Performance Status Intervention vs Comparison n	Stage at baseline Intervention vs Comparison n
Chemotherapy						
Frey, 1981 [30]	152 65 vs 87	*n* intervention/comparison under 50: 6/11 50–59: 31/40 60–69: 16/19 70 and over: 10/13 NR: 2/4	0	Intervention: White: 69.2% Nonwhite: 27.7% NR: 3.1%. Comparison: White: 67.8% Nonwhite: 32.2%	NR	**Invasion (neural,blood vessels, lymphatic)** 22 vs 28
Glimelius, 1996 [31]	53^a^ 29 vs 24	*Median (range)* Intervention 65 (43–74) Comparison 64 (48–75)	Intervention: *n* = 33 Comparison: *n* = 26	NR	NR	NR
Huguier, 2001 [32]	45 22 vs 23	*Mean (range)* Intervention: 64.7 (47–75) Comparison: 62.2 (36–77)	18 vs 74	NR	**WHO PS 0** 11 vs 4 **WHO PS 1** 7 vs 7 **WHO PS 2** 4 vs 12	**With Metastases** 11 vs 10
Mallinson, 1980 [33]	40 21 vs 19	*Median* Intervention: 63.4 Comparison: 66.9	43 vs 47	NR	NR	**With Metastases** 7 vs 8 **Local disease** 14 vs 11
The Gastrointestinal Tumor Study Group, 1979 [35]	89 29 vs 32 vs 28	*n* All participants: under 50: 17 50–59: 32 60–69: 30 70 and over: 9 age unknown = 1	Intervention 1 vs Intervention 2 vs Comparison 45 vs 47 vs 43	NR	Intervention 1 vs Intervention 2 vs Comparison **PS 0–1** 21 vs 16 vs 16 **PS 2–3** 8 vs 16 vs 12	NR (Unresectable locally advanced PC**)**
Xinopoulos, 2008 [36]	49 16 vs 33	*Mean (range)* Intervention: 66.5 (59–73) Comparison: 66.6 (58–73)	44 vs 45	NR	NR	**Metastatic** 6 vs 12
Ciuleanu, 2009 [38]	303 148 vs 155	*Median (range)* Intervention: 58 (27–78) Comparison: 57 (29–80)	39 vs 42	Intervention: White: 87% "Other": 13% Comparison: White: 87% "Other": 14%	**KPS 100** 23 vs 20 **KPS 90** 36 vs 48 **KPS 80** 62 vs 62 **KPS 70** 27 vs 25	NR (Metastatic pancreatic cancer)
Pelzer, 2011 [39]	46 23 vs 23	*Median (range)* Intervention: 60 (38–76) Comparison: 61 (34–80)	39 vs 35	NR	**KPS 70–80%** 12 vs 11 **KPS 90–100%** 11 vs 12 **median (range)** **KPS** 80 (70–100) vs 80 (70–100)	**M0** 6 vs 7 **M1** 17 vs 16
Palmer, 1994 [41]	43 23 vs 20	*Median (range)* Intervention: 61 (41–81) Comparison: 62 (41–81)	30 vs 25	NR	**WHO PS 0 or 1** 21 vs 19 **WHO PS 2** 2 vs 1	**With Metastases** 9 vs 10
Shinchi, 2002 [42]	31 16 vs 15	*Mean (SD)* Intervention: 62.9 (2.8) Comparison: 64.6 (4.0)	36 vs 67	NR	NR	Stage IVA
Takada, 1998 [43]	52 28 vs 24	*Mean (range)* Intervention: 62.8 (46–74) Comparison: 61.5 (43–74)	33 vs 37	NR	**PS 0–1** 19 vs 11 **PS 2–3** 9 vs 10 **Unclear** 0 vs 3	**Stage III** 1 vs 0 **Stage IV** 27 vs 24
Immunotherapy						
Oortgiesen, 2010 [44]	154 79 vs 75	NR	NR	NR	NR	NR **(Stage II, III or IV)**
Gilliam, 2012 [45]	154 79 vs 75	*Median (Range)* Intervention: 62 (40–83) Comparison: 62 (36–89)	51 vs 52	NR	**KPS 80–100** 49 vs 43 **KPS 60–70** 30 vs 31 **KPS < 60** 0 vs 1^b^	**Stage II/III** 20 vs 11 **Stage IV** 58 vs 63
Targeted/biological therapy						
Propper, 2014 [47]	207 104 vs 103	*Median (Range)* Intervention: 62 (33–90) Comparison: 57 (25–88)	43 vs 43	NR	**ECOG PS 0/1** 88 vs 86 **ECOG PS 2** 16 vs 17	**Locallly Advanced** 20 vs 15 **Metastatic** 84 vs 88

*NR* Not reported, *PS* Performance status, *KPS* Karnofsky PS, *ECOG* Eastern Cooperative Oncology Group

^a^Study also included participants with biliary tract cancer. Baseline information is not disaggregated

^b^At screening, 70 (30 at baseline, should not have been entered)

We included 14 RCTs assessing the effects of TAD vs either BSC, SC, placebo, or no treatment in patients with advanced PC [30–33, 35, 36, 38, 39, 41–45, 47]. The year of publication ranged from 1979 to 2014, with only two studies published in the last 10 years [45, 47]. Eight (57.1%) studies did not specify the sources of funding and nine (64.2%) did not report conflicts of interest.

##### Chemotherapy* versus No-TAD*

We included 11 studies for this comparison [30–33, 35, 36, 38, 39, 41–43]. The number of participants included in each study ranged from 31 to 303, totalling 903 participants.

The studies included participants with unresectable advanced PC [30, 32, 33, 35, 36, 39, 41–43], “non-curable PC” [31], or only metastatic PC [38]. Six studies provided baseline performance status (PS) details: two included participants with Karnofsky PS (KPS) > 70 [38, 39], two with WHO PS 0 to 2 [32, 41], and two with WHO PS 0 to 3 [35, 43] (Table 2). Three studies only provided this information in their eligibility criteria [31, 36, 42], and two studies did not inform baseline PS [30, 33].

Nine studies assessed 5-FU-based regimes [30–33, 35, 39, 41–43], one study assessed gemcitabine [36], and one glufosfamide [38]. Six studies evaluated the intervention as first line [30–33, 35, 36], two as second [38, 39] and three as non-specified lines of therapy [41–43]. For the control arms, seven studies described it as either BSC [31, 38, 39, 42] or SC [32, 33, 41]. Both groups received palliative surgery in three studies [30, 36, 43] and palliative radiotherapy in one study [35].

##### Immunotherapy versus No-TAD

We included two studies for this comparison [44, 45]. Results of the study by Oortgiesen et al. were only available as an abstract [44]. Each study included 154 participants, with unresectable advanced PC [44] or with advanced PC unwilling or unsuitable to receive chemotherapy [45].

One study planned to include participants with KPS ≥ 60, however, due to a screening error, it included one participant with KPS 30 at baseline in the control arm [45] and the other did not inform participants’ baseline PS [44] (Table 2).

One study tested a polyclonal antibody stimulator (PAS) vaccination, which elicits a specific and high-affinity anti-gastrin antibody and did not specify the line of therapy [44], while the other tested an anti-gastrin immunogen (G17DT) as first-line therapy [45]. In both studies, the control arm received a matching placebo.

##### Targeted/biological therapy versus No-TAD

We included one study for this comparison, which included 207 participants with unresectable locally advanced or metastatic PC. All participants had an ECOG PS 0 to 2 (Table 2). The study evaluated erlotinib without specifying the line of therapy and compared it to a matching placebo [47].

#### Risk of bias of included studies

We judged only one study to have a low risk of bias for all outcomes [47]. We judged four studies to have some concerns about risk of bias for all outcomes, mainly due to lack of information about allocation concealment and a pre-planned analysis [30, 32, 36, 44]. We judged four studies to have a high risk of bias for all outcomes, mainly due to deviations from intended interventions and issues arising from the randomisation process [31, 33, 38, 45]. Lastly, we judged five studies to have some concerns of risk of bias for survival outcomes, mainly due to lack of information about allocation concealment: however, for outcomes such as symptoms related to the disease, toxicity, and functional status, we judged them as having a high risk of bias, mainly due to concerns about differences in the measurement of the outcomes between groups [35, 39, 41–43] (See additional file 4 for the risk of bias per outcome in all the included studies).

### Effects of interventions

See Tables 3, 4 and 5 and appendices 5 and 6 for a summary of the results and certainty of evidence per outcome.

**Table 3:** Summary of findings for chemotherapy compared to no-TAD for advanced pancreatic cancer

**Outcomes**	**№ of participants** **(studies)** **Follow-up**	**Certainty of the evidence** **(GRADE)**	**Relative effect** **(95% CI)**	Anticipated absolute effects*	
				**Risk with no-TAD**	**Risk difference with chemotherapy**
Overall Survival (months)	122 (3 RCTs) [32, 39, 42]	⨁⨁◯◯ Low^a,b^	-	OS ranged from 2.5 to 7 months	MD 2.97 higher (1.23 higher to 4.7 higher)
Overall Survival (time to event)	647 (6 RCTs) [30, 32, 36, 38, 39, 43] 8 to 20 months	⨁◯◯◯ Very low^c,d,e^	HR 0.92 (0.74 to 1.16)	900 per 1,000 [death]	20 fewer per 1,000 (82 fewer to 31 more) [death]
Progression-Free Survival	303 (1 RCT) [38] 1 year	⨁◯◯◯ Very low^f,g^	HR 0.76 (0.57 to 1.05)	930 per 1,000 [progression]	63 fewer per 1,000 (150 fewer to 9 more) [progression]
Quality of Life assessed with: EORTC QLQ-C30—Higher scores mean better QoL	102 (2 RCTs) [31, 36] 4 to 6 months	⨁◯◯◯ Very low^h,i^	· Glimelius et al. categorised the results and reported favourable QoL in 11 (37.9%) participants in the chemotherapy group compared to 3 (12.5%) in the control group. Favourable QoL was defined as an unchanged high QoL or substantially improved scores for 4 months · Glimelius et al. additionally reported QoL-adjusted survival months (QASM), with a median of 4 in the intervention and 1 in the control group (*p* < 0.01) · Xinopoulos et al. reported superior scores in the intervention group only during the first month (*p* = 0.028). From the second to fourth month there were no differences and during the fifth and sixth months, patients in the control group had significantly higher scores compared to the intervention group (*p* = 0.010, 0.0003, respectively), as well as when considering the average score for all the weeks of follow-up (*p* = 0.0001)		
Functional Status assessed with: PS scores	83 (2 RCTs) [42, 43]	⨁◯◯◯ Very low^j,k^	· Shinchi et al. found a MD of 11.6 (95%CI 10.33, 12.87) in the monthly KPS score in favour of the chemotherapy group · Additionally, they found a MD of 4 (95%CI 3.5, 4.5) KPS-score-adjusted survival months, in favour of the chemotherapy group · Takada et al. did not find statistically significant differences in FS between the groups, with 4 (14%) participants in the intervention and 2 (8%) in the control group presenting an improvement in their performance status by at least one grade, after the effects of the operation had disappeared (*p* = 0.051)		
Severe Adverse Events assessed with: National Cancer Institute’s Common Toxicity Criteria (CTCAE), WHO criteria	452 (4 RCTs) [35, 38, 39, 42]	⨁◯◯◯ Very low^l,m,n^	· Ciuleanu et al. reported a higher occurrence of any grade 3, 4 or 5 adverse events in the chemotherapy group (RR: 1.12; 95%CI 0.78, 1.60) · Shinchi et al., Pelzer et al., and TGTSG did not find significant differences in specific grade 3, 4 or 5 adverse events between groups		
Symptoms related to the disease	110 o (4 RCTs) [33, 38, 41, 43]	⨁◯◯◯ Very low^k,p,q^	· Ciuleanu et al. found that a higher proportion of participants in the intervention group had no increase in pain for at least 2 consecutive cycles, compared to the control group (72% vs 44%; *p* = 0.02) · Mallinson et al. found that more participants in the intervention group experienced nausea compared to the control group (80.9% vs 43.8%; *p* = 0.04), but there were no differences for pain, vomiting and diarrhoea · Palmer et al. found no statistically significant differences between groups in the number of participants with case level depression and anxiety · Takada et al. found no statistically significant differences between groups on the number of participants with any improvement in symptoms with at least 2 kg body weight gain		
Number of hospital days assessed with: Adjusted per month of survival	31 (1 RCT) [42] median 6 to 13 months	⨁⨁◯◯ Low^k,r^	-	The mean number of hospital days was 19	MD 6.7 lower (8.3 lower to 5.1 lower)

^*^The risk in the intervention group (and its 95% confidence interval) is based on the assumed risk in the comparison group and the relative effect of the intervention (and its 95% CI)

We used data from Kaplan–Meier survival curves from the control groups of the trials included in the systematic review to estimate the baseline risk for OS and PFS. *CI* Confidence interval, *HR* Hazard Ratio, *MD* Mean difference, *RR* Risk ratio

GRADE Working Group grades of evidence

High certainty: we are very confident that the true effect lies close to that of the estimate of the effect. Moderate certainty: we are moderately confident in the effect estimate: the true effect is likely to be close to the estimate of the effect, but there is a possibility that it is substantially different. Low certainty: our confidence in the effect estimate is limited: the true effect may be substantially different from the estimate of the effect. Very low certainty: we have very little confidence in the effect estimate: the true effect is likely to be substantially different from the estimate of effect

Explanations

^a^We downgraded one level due to some concerns of risk of bias of the included studies, mainly due to lack of information about allocation concealment

^b^We downgraded one level due to inconsistency. Although statistical heterogeneity was high (I2: 94%), all studies favoured the chemotherapy arm

^c^We downgraded one level due to some concerns of risk of bias of the included studies, mainly due to lack of information about allocation concealment and a pre-planned analysis. We judged Ciuleanu et al. to have high risk of bias due to deviation from the interventions, however, through sensitivity analysis, we found that this study did not impact the overall estimator in the direction of the effect nor precision, so we did not downgrade more levels

^d^We downgraded one level due to inconsistency. There was moderate statistical heterogeneity (I2: 46%)

^e^We downgraded three levels due to imprecision. The confidence interval shows both benefit and considerable harm

^f^We downgraded two levels due to high risk of bias, because of deviations from the intended interventions. There was additionally lack of information about allocation concealment

^g^We downgraded two levels due to imprecision. The confidence interval shows both benefit and harm

^h^We downgraded two levels due to high risk of bias due to suspected differences in the measurement of outcomes between groups (Glimelius et al. and Xinopoulos et al.), deviation from intended interventions (Glimelius et al.) and lack of information about allocation concealment (Xinopoulos et al.)

^i^We downgraded one level due to inconsistency. Studies show contradicting results, one favouring the chemotherapy arm and the other, the control arm

^j^We downgraded two levels due to high risk of bias due to suspected differences in the measurement of outcomes between groups (Shinchi et al. and Takada et al.), lack of information about allocation concealment (Shinchi et al.) and suspected selective reporting (Takada et al.)

^k^We downgraded one level due to imprecision. Studies with small sample sizes

^l^We downgraded two levels due to high risk of bias of all studies mainly due to suspected differences in the measurement of the outcome between groups

^m^We downgraded one level due to inconsistency. Studies show contradicting results, one favouring the control arm and the others showing no difference between groups

^n^We downgraded one level due to imprecision. Studies with small sample size and low number of events that resulted in imprecise estimates

^o^One study (Ciuleanu et al.) did not report the number of participants assessed for this outcome

^p^We downgraded two levels due to high risk of bias of all studies, mainly due to suspected differences in the measurement of the outcome between groups (all), some concerns about selective reporting (Ciuleanu et al., Mallinson et al. and Takada et al.) and for deviation from the intended interventions (Ciuleanu et al. and Mallinson et al.)

^q^We downgraded two levels due to inconsistency. Studies show contradicting results, some favouring the control arm and some showing no difference between groups. Additionally, studies used different scales to assess this outcome

^r^We downgraded one level due to some concerns about risk of bias because of lack of information about allocation concealment

**Table 4:** Summary of findings for immunotherapy compared to no-TAD for advanced pancreatic cancer

Immunotherapy compared to no-TAD for advanced pancreatic cancer			
**Outcomes**	**№ of participants** **(studies)** **Follow-up**	**Certainty of the evidence** **(GRADE)**	**Narrative results**
Overall Survival	304 (2 RCTs) [44, 45] up to 400 days	⨁◯◯◯ Very low^a,b^	· Gilliam et al. reported a median of 100 days in the intervention and 78 days in the control group, for patients with PC stage IV, and of 193 vs 239 days for patients with PC stage II/III (HR 0.75; 95%CI 0.51, 1.10) · Oortgiesen et al. reported a median of 150 days in the intervention group and 84 days for the control group
Functional Status assessed with: Time to deterioration of KPS (below 60)	152 (1 RCT) [45]	⨁◯◯◯ Very low^c,d^	· Gilliam et al. reported the median time to deterioration of 138 days for the immunotherapy group and 78 days for the control group (*p* = 0.058)

GRADE Working Group grades of evidence

High certainty: we are very confident that the true effect lies close to that of the estimate of the effect

Moderate certainty: we are moderately confident in the effect estimate: the true effect is likely to be close to the estimate of the effect, but there is a possibility that it is substantially different

Low certainty: our confidence in the effect estimate is limited: the true effect may be substantially different from the estimate of the effect

Very low certainty: we have very little confidence in the effect estimate: the true effect is likely to be substantially different from the estimate of effect

Explanations

^a^We downgraded two levels due to high risk of bias arising from problems in the randomisation process (Gilliam et al.) and some concerns due to lack of information about allocation concealment, deviation from interventions and selective reporting (Oortgiensen et al.)

^b^We downgraded two levels due to imprecision. The confidence interval shows both benefit and harm

^c^We downgraded two levels due to high risk of bias arising from problems in the randomisation process

^d^We downgraded two levels due to imprecision. Only one study assessed this outcome with a small sample size resulting in imprecise estimators

**Table 5:** Summary of findings for targeted/biological therapy compared to no-TAD for advanced pancreatic cancer

**Outcomes**	**№ of participants** **(studies)** **Follow-up**	**Certainty of the evidence** **(GRADE)**	**Relative effect** **(95% CI)**	Anticipated absolute effects*	
				**Risk with No-TAD**	**Risk difference with targeted/biological therapy**
Overall Survival	207 (1 RCT) [47] 2 years	⨁⨁◯◯ Low^a^	HR 1.04 (0.77 to 1.39)	900 per 1,000 [death]	9 more per 1,000 (70 fewer to 59 more) [death]
Progression-Free Survival	207 (1 RCT) [47] 3 years	⨁⨁◯◯ Low^a^	HR 0.83 (0.63 to 1.10)	930 per 1,000 [progression]	40 fewer per 1,000 (117 fewer to 16 more) [progression]
Adverse events assessed with: patients with one or more treatment related serious adverse event (grade 3,4,5 CTCAE)	207 (1 RCT) [47]	⨁⨁⨁◯ Moderate^b^	RR 5.45 (1.24 to 23.97)	19 per 1,000	86 more per 1,000 (5 more to 446 more)

^*^The risk in the intervention group (and its 95% confidence interval) is based on the assumed risk in the comparison group and the relative effect of the intervention (and its 95% CI)

We used data from Kaplan–Meier survival curves from the control groups of the trials included in the systematic review to estimate the baseline risk for OS and PFS

*CI* Confidence interval, *HR* Hazard Ratio, *MD* Mean difference, *RR* Risk ratio

GRADE Working Group grades of evidence

High certainty: we are very confident that the true effect lies close to that of the estimate of the effect

Moderate certainty: we are moderately confident in the effect estimate: the true effect is likely to be close to the estimate of the effect, but there is a possibility that it is substantially different

Low certainty: our confidence in the effect estimate is limited: the true effect may be substantially different from the estimate of the effect

Very low certainty: we have very little confidence in the effect estimate: the true effect is likely to be substantially different from the estimate of effect

Explanations

^a^We downgraded two levels due to imprecision. Single study with a small sample size and the confidence interval shows both benefit and harm

^b^We downgraded one level due to imprecision. Single study with a small sample size and low number of events

#### Chemotherapy versus No-TAD

Eight studies reported a longer median or mean OS in the group that received chemotherapy [31–33, 35, 38, 39, 41, 42], while two reported a longer OS in the no-TAD group [30, 36]. Only three studies provided sufficient data for meta-analysis for OS as a continuous outcome and showed that chemotherapy may slightly increase OS (MD: 2.97 months (95%CI 1.23, 4.70); low certainty) [32, 39, 42] (Additional file 5.1).

Six studies provided sufficient data to calculate HR for OS, with 647 participants analysed. The pooled analysis showed chemotherapy may have little to no effect on OS (HR: 0.92 (95%CI 0.74, 1.16); very low certainty) (Additional file 5.1). Pooled analysis of seven studies showed that chemotherapy resulted in an absolute mortality risk reduction of 253 per 1,000 patients at 6-months (RR: 0.67 (95%CI 0.48, 0.94); very low certainty) and of 135 per 1,000 patients at 12-months (RR: 0.86 (95%CI: 0.76, 0.98; very low certainty)(Additional file 5.2). Lastly, only one study assessed PFS and reported results in favour of chemotherapy, however inconclusive (HR: 0.76 (95%CI 0.57, 1.05); very low certainty) [38].

QoL was assessed by only two studies. Glimelius et al. reported results in favour of chemotherapy over a 4-month period [31], while Xinopoulos et al. found that in a 6-month period the no-TAD group had better results [36]. Two studies assessed participants’ FS and reported results in favour of chemotherapy [42, 43]. Additionally, seven studies reported toxicity [32, 35, 36, 38, 39, 41, 42], but only four did so in both groups allowing for comparison (Additional file 5.3). Ciuleanu et al. reported a higher occurrence of toxicities in the chemotherapy group [38], while the other three reported no significant differences between groups for each adverse event [35, 39, 42]. Lastly, four studies assessed symptoms related to the disease using different scales to measure pain [33, 38], gastrointestinal symptoms [33], depression and anxiety [41], and improvement of overall symptomatology [43] and reported contradicting results (Additional file 5.4). Overall, the evidence is very uncertain about the effect of chemotherapy on QoL, FS, toxicity and symptoms related to the disease.

Only one study assessed the number of hospital days and reported a lower mean in the control group (99.3 versus 115.7 days). However, after adjusting per month of survival, the chemotherapy group presented fewer hospital days (MD: -6.7 (-8.3, -5.1); low certainty) [42].

We did not find studies that assessed QoD in both groups. Only one study assessed an aspect of QoD in the chemotherapy group and reported that 15 out of 16 participants in the intervention group did not receive gemcitabine in the last 2–3 weeks before death when their condition was very poor. The other participant had previously requested treatment discontinuation [36].

#### Immunotherapy versus No-TAD

The two studies assessing immunotherapy reported results for OS in favour of the intervention, with a median of 22 to 66 days of advantage [44, 45], with only one reporting a HR of 0.75 (95%CI 0.51, 1.10); very low certainty. Only one study assessed FS and reported longer time to deterioration of KPS (below 60) in the immunotherapy group [45]. Overall, the evidence is very uncertain about the effect of immunotherapy in OS and FS.

We did not find studies that assessed or adequately reported the following outcomes: PFS, QoL, toxicity, symptoms related to the disease, admissions to hospital, and QoD.

#### Targeted/biological therapy versus No-TAD

The only study addressing this comparison reported that erlotinib may result in little to no difference in OS (HR: 1.04 (95%CI 0.77,1.39); low certainty) and it may increase PFS (HR: 0.83 (95%CI 0.63, 1.10); low certainty). However, it probably results in an increase in treatment related serious adverse events (RR. 5.54 (95%CI 1.24, 23.97); moderate certainty) (Additional file 5.3) [47].

We did not find studies that assessed or adequately reported the following outcomes: QoL, FS, symptoms related to the disease, admissions to hospital and QoD.

## Discussion

Our SR identified, evaluated, and summarised the evidence of a total of 14 RCTs that assessed the efficacy of TADs versus SC or no treatment for patients with advanced PC. We identified very few RCTs that compared TADs, either chemotherapy (*n* = 11), immunotherapy (*n* = 2), or targeted/biological therapies (*n* = 1) versus no oncological treatment (using as comparator usual supportive care or placebo or no treatment), with most assessing only first-line therapies. Besides the sparsity in the number of trials, there are several serious drawbacks to consider in order to interpret the results correctly. First, the overall certainty of evidence was low or very low, due to many concerns of risk of bias, inconsistency in the results, and imprecision of the effect estimates. Additionally, most studies lack transparency regarding potential conflicts of interest. Given all these limitations, it is remarkable that there are only two RCTs conducted in the last ten years, since this is still a critical question with an important level of uncertainty.

Second, outcomes different from survival are seldom measured or reported —even when they are undeniably relevant in this context. Patients with advanced PC often present a substantial symptom burden (including pain, tiredness and lack of energy) which affects their functionality, daily life activities [49], and ultimately constitutes one of the main reasons for TADs discontinuation [50]. Moreover, a recent RCT confirmed that the integration of routine symptom monitoring during cancer treatment improves survival outcomes [51]. Unfortunately, the included RCTs in our study did not systematically assess potential changes in symptoms control or quality of life throughout the course of treatments.

Third, we could not include all the data provided by the primary studies to assess toxicity either, due to several reasons. In some cases, authors provided data only for the experimental arm, with no information given for the control group. Others did not assess the adverse events grouped by severity, reporting the results extensively disaggregated. Finally, in some reports it was unclear whether the unit of analysis were the patients or the events (e.g. if two adverse events in one patient were counted as “2” or “1”). We collected data only from those primary studies whose reports were appropriate for our predefined methods, but there is a large room for improvement when reporting toxicity form RCTs.

Fourth, almost all studies included participants with a PS from 0 to 2, which is consistent with the population considered in guidelines’ recommendations for the use of TAD for advanced PC [7, 9, 52]. As we did not find studies assessing this comparison for patients with PS 3 or higher, this lack of evidence related to more advanced patients would be an additional argument for rigorously assessing the performance status of all PC patients in clinical practice to avoid treating those with a poorer prognosis and therefore, with increasing chances to produce them more harms than benefits. This is why there is a progressive recognition about the importance of a valid evaluation of patients’ experience through patient-reported outcome (PRO) instruments. Instruments such as the EORTC QLQ-C30/PAN26 have been deemed highly relevant by patients since it measures most aspects of their experience with the disease and treatment [49]. While it is a great accomplishment that some newer studies are including these measures, it is regrettable that they were not used when comparing TADs to less aggressive treatments whose aim was to improve cancer-related symptomatology and QoL, such as SC [53].

Given all the above-mentioned limitations, the quantitative results from our SR must be interpreted with caution. Chemotherapy may result in a slight increase in OS of about 3 months and fewer hospital days compared to SC (about 1 week), while targeted/biological therapies may provide little or no difference in OS and a slight increment on PFS, at the expense of more treatment-related serious adverse events. As already said, there is an important uncertainty about the effects of chemotherapy in PFS, QoL and FS, and even more of immunotherapy in OS and FS. Moreover, as there are no RCTs that have assessed the effects of treating advanced PC beyond a second-line therapy, this lack of evidence should be another criterion for establishing treatment limits.

Given the evidence gaps and the low certainty of evidence of our findings, the assessment of the effects of TADs when compared to SC alone for patients with advanced PC may still be considered appropriate. Some clinicians could justify their use of TADs in advanced PC patients based in the superiority of some regimens over others, since chemotherapy is already considered the standard of care [7, 9]. But others can argue that critical and important outcomes for decision making have not been sufficiently studied and that guidelines’ recommendations of TADs are based on limited evidence and a partial evaluation of their effects. Therefore, it would be reasonable and ethical to conduct more and better RCTs considering BSC with no TAD as the control arm in PC patients with an advanced disease. The information obtained from these studies would certainly enhance the future decision-making process.

This SR has several strengths. We conducted an exhaustive search in six databases, as well as citation search to find all relevant studies. We have also conducted selection, data extraction and quality assessment by two reviewers independently, and we present the results with the respective certainty of evidence assessment using the GRADE guidance.

Although our review did not focus on comparing different schemes of TADs with each other, it really challenges the appropriateness of current recommendations of TADs and the way in which they have been formulated. Therefore, clinicians should continuously provide advanced PC patients with all relevant information about prognosis, treatment options including BSC as a reasonable alternative, and other aspects of care, thus advocating for patient’s autonomy [9]. In the case of advanced PC patients, it is crucial to assume that their very poor survival prognosis at short term basis would be only slightly improved with any TAD, if successful, and this must be balanced with the risks associated to the therapies that could worsen their QoL. If they are aware of this scenario, some patients could reasonably prefer to receive only the best possible SC and give priority to maintain a good QoL until the end of their lives.

## Conclusions

This review found that the evidence driven from RCTs is very uncertain about whether the benefits provided by TADs are greater than their associated harms in patients with advanced PC. When the first chemotherapy lines have failed, there is no evidence to propose further TADs to patients unless accepting their inclusion in a trial. In contrast, BSC is an appropriate alternative to be offered, especially if their functional status is poor or the disease is very advanced. Future research should assess the impact of TADs on all patient-important outcomes, thus providing relevant information to involve patients in establishing their goals of care.

### Supplementary Information

**Additional file 1. **Search strategy for PUBMED and CENTRAL.

**Additional file 2. **Excluded reports.

**Additional file 3. **Characteristics of included studies.

**Additional file 4. **Judgements for each risk of bias domain for included studies.

**Additional file 5. **Results for chemotherapy and targeted/biological therapy versus no-TAD per outcome.

**Additional file 6. **Summary of findings for chemotherapy compared to no-TAD for advanced pancreatic cancer.

## Data Availability

The protocol of the current study is available in the OSF repository, https://osf.io/7chx6/, (DOI https://doi.org/10.17605/OSF.IO/7CHX6). Search strategies are included in the supplementary information files.

## Abbreviations

BSC: Best supportive care
CI: Confidence interval
EoL: End-of-life
ECOG: Eastern Cooperative Oncology Group
FS: Functional status
GRADE: Grading of Recommendations Assessment, Development, and Evaluation
HR: Hazard ratio
KPS: Karnofsky Performance Status
MD: Mean Difference
OS: Overall Survival
PC: Pancreatic Cancer
PFS: Progression-free Survival
PRISMA: Preferred Reporting Items for Systematic reviews and Meta-Analyses
PS: Performance status
QoL: Quality of Life
QoD: Quality of Death
RCT: Randomised Clinical Trial
RoB: Risf of Bias
RR: Risk Ratio
SC: Supportive Care
SoF: Summary of Findings
SR: Systematic Review
TAD: Treatment with Anticancer Drugs
