# Supplementary material for: Treatment with anticancer drugs for advanced pancreatic cancer: a systematic review
Source: BMC Cancer. 2023 Aug 12;23:748. doi: 10.1186/s12885-023-11207-4 (PMC10422698; doi:10.1186/s12885-023-11207-4)
Supplement: Supplementary file 1 — Additional file 1. Search strategy for PUBMED and CENTRAL. [file 12885_2023_11207_MOESM1_ESM.docx]

**Search strategy for PUBMED and CENTRAL**

PUBMED

| #1 | "Pancreatic Neoplasms"[Mesh] |
| --- | --- |
| #2 | ((pancreas*[Title] OR pancreatic*[Title]) AND (cancer*[Title] OR carcinom*[Title] OR neoplasm*[Title] OR tumor*[Title] OR tumour*[Title] OR malignan*[Title] OR adenocar*[Title] OR oncolog*[Title])) |
| #3 | (#1 OR #2) |
| #4 | ("Palliative Care"[Mesh] OR "Terminal Care"[Mesh] OR "Neoplasm Metastasis"[Mesh]) |
| #5 | (palliative*[Title/Abstract] OR end of life*[Title/Abstract] OR end of live*[Title/Abstract] OR terminal*[Title/Abstract] OR metasta*[Title/Abstract] OR BSC[Title/Abstract] OR supportive care*[Title/Abstract] OR advanced*[Title/Abstract] OR unresect*[Title/Abstract] OR irresect*[Title/Abstract] OR nonresect*[Title/Abstract] OR non resect*[Title/Abstract] OR inopera*[Title/Abstract] OR unopera*[Title/Abstract] OR nonopera*[Title/Abstract] OR non opera*[Title/Abstract] OR non-opera*[Title/Abstract] OR stage IV[Title/Abstract]) |
| #6 | (#4 OR #5) |
| #7 | (#3 AND #6) |
| #8 | ("Antineoplastic Protocols"[Mesh] OR "Chemoradiotherapy"[Mesh] OR "Induction Chemotherapy"[Mesh] OR "Maintenance Chemotherapy"[Mesh] OR "Consolidation Chemotherapy"[Mesh]) |
| #9 | (antineoplastic*[Title] OR antineoplasic*[Title] OR chemotherap*[Title] OR chemoradiotherap*[Title] OR radiochemotherap*[Title] OR carboplatin*[Title] OR cisplatin*[Title] OR oxaliplatin*[Title] OR platin*[Title] OR fluorouracil*[Title] OR 5-FU[Title] OR capecitabine*[Title] OR docetaxel*[Title] OR taxotere[Title] OR epirubicin*[Title] OR irinotecan*[Title] OR onivyde[Title] OR paclitaxel*[Title] OR abraxane[Title] OR trifluridine*[Title] OR tipiracil*[Title] OR lonsurf[Title] OR gemcitabine*[Title] OR gemzar[Title] OR mitomycin*[Title]) |
| #10 | (#8 OR #9) |
| #11 | ("Molecular Targeted Therapy"[Mesh] OR "Antibodies, Monoclonal"[Mesh] OR "Cancer Vaccines"[Mesh]) |
| #12 | (Target*[Title] OR antibod*[Title] OR immunotherap*[Title] OR vaccine[Title] OR vaccines[Title] OR vaccination[Title] OR tyrosine kinase inhibit*[Title] OR trastuzumab[Title] OR herceptin[Title] OR bevacizumab[Title] OR rilotumumab[Title] OR onartuzumab[Title] OR ramucirumab[Title] OR cyramza[Title] OR cetuximab[Title] OR panitumumab[Title] OR nimotuzumab[Title] OR claudiximab[Title] OR apatinib[Title] OR lapatinib[Title] OR regorafenib[Title] OR stivarga[Title] OR everolimus[Title] OR nivolumab[Title] OR opdivo[Title] OR pembrolizumab[Title] OR keytruda[Title] OR avelumab[Title] OR durvalumab[Title] OR ipilimumab[Title] OR checkpoint inhibit*[Title] OR cabozantinib[Title] OR cabometyx[Title] OR lenvatinib[Title] OR lenvima[Title] OR sorafenib[Title] OR nexavar[Title] OR sunitinib[Title] OR sutent[Title] OR erlotinib[Title] OR tarceva[Title] OR doxorubicin[Title]) |
| #13 | (#11 OR #12) |
| #14 | (#10 OR #13) |
| #15 | (#7 AND #14) |
| #16 | (animals [mh] NOT humans [mh]) |
| #17 | (#15 NOT #16) |
| #18 | randomized controlled trial[pt] OR controlled clinical trial[pt] OR randomized[tiab] OR placebo[tiab] OR clinical trials as topic[mesh:noexp] OR randomly[tiab] OR trial [ti] |
| #19 | (#17 AND #18) |
| #20 | 2019/12/01:2022/04/26[pdat] |
| #21 | (#19 AND #20) |

COCHRANE CENTRAL REGISTER OF CONTROLLED TRIALS

ID Search

#1 MeSH descriptor: [Pancreatic Neoplasms] explode all trees

#2 pancreas*:ti,ab NEAR/5 (cancer* OR carcinom* OR neopla* OR tumor* OR tumour* OR malignan* OR adenocar* OR oncolog*):ti,ab

#3 #1 OR #2

#4 MeSH descriptor: [Palliative Care] explode all trees

#5 MeSH descriptor: [Terminal Care] explode all trees

#6 MeSH descriptor: [Neoplasm Metastasis] explode all trees

#7 (palliative* OR “end of life” OR “end of live” OR terminal* OR metasta* OR BSC OR “supportive care” OR advanced* OR unresect* OR irresect* OR nonresec* OR (non NEXT resec*) OR inopera* OR unopera* OR nonopera* OR (non NEXT opera*) OR (stage NEXT IV)):ti,ab

#8 #4 OR #5 OR #6 OR #7

#9 #3 AND #8

#10 MeSH descriptor: [Antineoplastic Protocols] explode all trees

#11 MeSH descriptor: [Chemoradiotherapy] explode all trees

#12 MeSH descriptor: [Induction Chemotherapy] explode all trees

#13 MeSH descriptor: [Maintenance Chemotherapy] explode all trees

#14 MeSH descriptor: [Consolidation Chemotherapy] explode all trees

#15 (antineoplastic* OR antineoplasic* OR chemotherap* OR chemoradiotherap* OR radiochemotherap* OR carboplatin* OR cisplatin* OR oxaliplatin* OR platin* OR fluorouracil* OR (5 NEXT FU) OR capecitabine* OR docetaxel* OR taxotere OR epirubicin* OR irinotecan* OR onivyde OR paclitaxel* OR abraxane OR trifluridine* OR tipiracil* OR lonsurf OR gemcitabine* OR gemzar OR mitomycin*):ti

#16 #10 OR #11 OR #12 OR #13 OR #14 OR #15

#17 MeSH descriptor: [Molecular Targeted Therapy] explode all trees

#18 MeSH descriptor: [Antibodies, Monoclonal] explode all trees

#19 MeSH descriptor: [Cancer Vaccines] explode all trees

#20 (target* OR antibod* OR immunotherap* OR vaccine OR vaccines OR vaccination OR (tyrosine NEXT kinase NEXT inhibit*) OR trastuzumab OR herceptin OR bevacizumab OR rilotumumab OR onartuzumab OR ramucirumab OR cyramza OR cetuximab OR panitumumab OR nimotuzumab OR claudiximab OR apatinib OR lapatinib OR regorafenib OR stivarga OR everolimus OR nivolumab OR opdivo OR pembrolizumab OR keytruda OR avelumab OR durvalumab OR ipilimumab OR (checkpoint NEXT inhibit*) OR (check NEXT point NEXT inhibit*) OR cabozantinib OR cabometyx OR lenvatinib OR lenvima OR sorafenib OR nexavar OR sunitinib OR sutent OR erlotinib OR tarceva OR doxorubicin):ti

#21 #17 OR #18 OR #19 OR #20

#22 #16 OR #21

#23 #9 AND #22

#24 #9 AND #22

with Cochrane Library publication date from Dec 2019 to Apr 2022
