# Supplementary material for: Treatment with anticancer drugs for advanced pancreatic cancer: a systematic review
Source: BMC Cancer. 2023 Aug 12;23:748. doi: 10.1186/s12885-023-11207-4 (PMC10422698; doi:10.1186/s12885-023-11207-4)
Supplement: Supplementary file 2 — Additional file 2. Excluded reports. [file 12885_2023_11207_MOESM2_ESM.docx]

**Excluded reports**

| **Study ID** | **Reason for exclusion** | **Reference** |
| --- | --- | --- |
| Zhou 2022 | Wrong intervention | Zhou, L., Yang, H., Xie, L., Sun, J., Qian, J., & Zhu, L. (2022). Comparison of Image-Guided Iodine-125 Seed Interstitial Brachytherapy and Local Chemotherapy Perfusion in Treatment of Advanced Pancreatic Cancer. Journal of investigative surgery : the official journal of the Academy of Surgical Research, 35(1), 1–6. https://doi.org/10.1080/08941939.2020.1805057 |
| Reni 2013 | Wrong population | Reni, M., Cereda, S., Milella, M., Novarino, A., Passardi, A., Mambrini, A., Di Lucca, G., Aprile, G., Belli, C., Danova, M., Bergamo, F., Franceschi, E., Fugazza, C., Ceraulo, D., & Villa, E. (2013). Maintenance sunitinib or observation in metastatic pancreatic adenocarcinoma: a phase II randomised trial. European journal of cancer (Oxford, England : 1990), 49(17), 3609–3615. https://doi.org/10.1016/j.ejca.2013.06.041 |
| Reni 2012 |  | Reni, M., Cereda, S., Milella, M., Novarino, A., Passardi, A., Mambrini, A., Di Lucca, G., Ferrari, L., Belli, C., Danova, M., Bergamo, F., Franceschi, E., Rovati, B., Fugazza, C., Ceraulo, D., & Villa, E. (2012). Maintenance sunitinib (MS) or observation (O) in metastatic pancreatic adenocarcinoma (MPA): Clinical and translational results of a phase II randomized trial (NCT00967603) [Abstract from 2012 ASCO Annual Meeting]. Journal of Clinical Oncology, 30(15_suppl), 4017–4017. https://doi.org/10.1200/jco.2012.30.15_suppl.4017 |
| Golan 2019 | Wrong population | Golan, T., Hammel, P., Reni, M., Van Cutsem, E., Macarulla, T., Hall, M. J., Park, J. O., Hochhauser, D., Arnold, D., Oh, D. Y., Reinacher-Schick, A., Tortora, G., Algül, H., O'Reilly, E. M., McGuinness, D., Cui, K. Y., Schlienger, K., Locker, G. Y., & Kindler, H. L. (2019). Maintenance Olaparib for Germline BRCA-Mutated Metastatic Pancreatic Cancer. The New England journal of medicine, 381(4), 317–327. https://doi.org/10.1056/NEJMoa1903387 |
| Hochhauser 2020 |  | Hochhauser, D., Kindler, H., Hammel, P., Reni, M., Van Cutsem, E., Macarulla, T., Hall, M. J., Park, J. O., Arnold, D., Oh, D. -Y., Reinacher-Schick, A., Tortora, G., Algül, H., O'Reilly, E. M., McGuinness, D., Cui, K., Schlienger, K., Locker, G., & Golan, T. (2020). Assessing clinical benefit of olaparib maintenance treatment in subgroups of patients with germline BRCA mutation (gBRCAm) and metastatic pancreatic cancer: Phase III POLO trial [Abstract]. Annals of Oncology, 31(4_suppl), S940–S941. <https://doi.org/10.1016/j.annonc.2020.08.2010> |
| Hall 2020 |  | Hall, M. J., Golan, T., Hammel, P., Reni, M., Van Cutsem, E., Macarulla, T., Park, J. O., Hochhauser, D., Arnold, D., Oh, D. -Y., Reinacher-Schick, A. C., Tortora, G., Algül, H., O'Reilly, E. M., McGuinness, D., Cui, K., Joo, S., Yoo, H.K., Patel, N., & Kindler, H. L. (2020). Pancreatic cancer (PaC)-specific health-related quality of life (HRQoL) with maintenance olaparib (O) in patients (pts) with metastatic (m) PaC and a germline BRCA mutation (gBRCAm): Phase III POLO trial [Abstract from 2020 Gastrointestinal Cancers Symposium]. Journal of Clinical Ongology, 38(4_suppl), 648–648. <https://doi.org/10.1200/JCO.2020.38.4_suppl.648> |
| Schwartz 2020 |  | Schwartz, L. H., Kindler, H. L., Hammel, P., Reni, M., Van Cutsem, E., Macarulla, T., Hall, M.J., Oh Park, J., Hochhauser, D., Arnold, D., Oh, D. -Y., Reinacher-Schick, A., Tortora, G., Alguel, H., O'Reilly, E. M., Fromageau, J., Ghiorghiu, D. C., McGuinness, D., Locker, G. Y., & Golan, T. (2020). POLO: Radiologic assessment of the impact of maintenance olaparib in patients (pts) with metastatic pancreatic cancer (mPaC) [Abstract from 2020 ASCO Annual Meeting]. Journal of Clinical Oncology, 38(15_suppl). <https://doi.org/10.1200/JCO.2020.38.15_suppl.e16800> |
| Yoo 2020 |  | Yoo, H. K., Kindler, H. L., McCutcheon, S., McGuinness, D., Patel, N., Hettle, R., Goodbody, R., Joo, S., Locker, G. Y., & Golan, T. (2020). POLO: Quality-adjusted (QA) progression-free survival (PFS) and patient (pt)-centered outcomes with maintenance olaparib in pts with metastatic pancreatic cancer (mPaC) [Abstract from 2020 ASCO Annual Meeting]. Journal of Clinical Oncology, 38(15_suppl), 4626–4626. <https://doi.org/10.1200/JCO.2020.38.15_suppl.4626> |
| Hammel 2019 |  | Hammel, P., Kindler, H. L., Reni, M., Van Cutsem, E., Macarulla Mercade, T., Hall, M. J., Park, J. O., Hochhauser, D., Arnold, D., Oh, D. -Y., Reinacher-Schick, A., Tortora, G., Algül, H., O'Reilly, E. M., McGuinness, D., Cui, K. Y., Joo, S., Yoo, H. K., Patel, N., & Golan, T. (2019). POLO: Health-related quality of life (HRQoL) of olaparib maintenance treatment versus placebo in patients with a germline BRCA mutation and metastatic pancreatic cancer (mPC) [Abstract]. Annals of Oncology, 30(5_suppl), V254–V255. <https://doi.org/10.1093/annonc/mdz422.004> |
| Hammel 2019 |  | Hammel, P., Kindler, H. L., Reni, M., Van Cutsem, E., Macarulla, T., Hall, M. J., Park, J. O., Hochhauser, D., Arnold, D., Oh, D. Y., Reinacher-Schick, A., Tortora, G., Algül, H., O'Reilly, E. M., McGuinness, D., Cui, K. Y., Joo, S., Yoo, H. K., Patel, N., Golan, T., … POLO Investigators (2019). Health-related quality of life in patients with a germline BRCA mutation and metastatic pancreatic cancer receiving maintenance olaparib. Annals of oncology : official journal of the European Society for Medical Oncology, 30(12), 1959–1968. https://doi.org/10.1093/annonc/mdz406 - Golan, |
| Golan 2020 |  | Golan T., Kindler, H. L., Park, J. O., Reni, M., Macarulla, T., Hammel, P., Van Cutsem, E., Arnold, D., Hochhauser, D., McGuinness, D., Locker, G. Y., Goranova, T., Schatz, P., Liu, Y. Z., & Hall, M. J. (2020). Geographic and Ethnic Heterogeneity of Germline BRCA1 or BRCA2 Mutation Prevalence Among Patients With Metastatic Pancreatic Cancer Screened for Entry Into the POLO Trial. Journal of clinical oncology : official journal of the American Society of Clinical Oncology, 38(13), 1442–1454. https://doi.org/10.1200/JCO.19.01890 |
| NCT02184195 |  | ClinicalTrials.gov Identifier: NCT02184195. Olaparib in gBRCA Mutated Pancreatic Cancer Whose Disease Has Not Progressed on First Line Platinum-Based Chemotherapy (POLO) |
| NCT04300114 | Wrong population | ClinicalTrials.gov Identifier: NCT04300114. A Study of Maintenance Treatment With Fluzoparib in gBRCA/PALB2 Mutated Pancreatic Cancer Whose Disease Has Not Progressed on First Line Platinum-Based Chemotherapy |
| NCT00428597 | Wrong population | ClinicalTrials.gov Identifier: NCT00428597. A Study Of Sunitinib Compared To Placebo For Patients With Advanced Pancreatic Islet Cell Tumors |
| Smeenk 2005 | Wrong study design | Smeenk, H. G., de Castro, S. M., Jeekel, J. J., Kazemier, G., Busch, O. R., Incrocci, L., Erdmann, J., Hop, W. C., Gouma, D. J., & van Eijck, C. H. (2005). Locally advanced pancreatic cancer treated with radiation and 5-fluorouracil: a first step to neoadjuvant treatment?. Digestive surgery, 22(3), 191–197. https://doi.org/10.1159/000087973 |
| Takasawa 2006 | Wrong study design | Takasawa, O., Fujita, N., Kobayashi, G., Noda, Y., Ito, K., & Horaguchi, J. (2006). Endoscopic biliary drainage for patients with unresectable pancreatic cancer with obstructive jaundice who are to undergo gemcitabine chemotherapy. World journal of gastroenterology, 12(45), 7299–7303. https://doi.org/10.3748/wjg.v12.i45.7299 |
| Tada 2008 | Wrong study design | Tada, M., Arizumi, T., Nakai, Y., Sasaki, T., Kogure, H., Togawa, O., Matsubara, S., Tsujino, T., Hirano, K., Sasahira, N., Isayama, H., Kawabe, T., & Omata, M. (2008). Efficacy of gemcitabine for locally advanced pancreatic cancer: comparison with 5-fluorouracil-based chemoradiotherapy. Chemotherapy, 54(4), 302–308. https://doi.org/10.1159/000151226 |
| Fujino 2008 | Wrong study design | Fujino, Y., Sakai, T., & Kuroda, Y. (2008). Palliative pancreatectomy with postoperative gemcitabine for patients with advanced pancreatic cancer. Journal of gastroenterology, 43(3), 233–238. https://doi.org/10.1007/s00535-007-2147-4 |
| Nakai 2008 | Wrong study design | Nakai, Y., Isayama, H., Kawabe, T., Tsujino, T., Yoshida, H., Sasaki, T., Tada, M., Arizumi, T., Yagioka, H., Kogure, H., Togawa, O., Ito, Y., Matsubara, S., Hirano, K., Sasahira, N., & Omata, M. (2008). Efficacy and safety of metallic stents in patients with unresectable pancreatic cancer receiving gemcitabine. Pancreas, 37(4), 405–410. https://doi.org/10.1097/MPA.0b013e3181706d93 |
| Mukherjee 2008 | Wrong study design | Mukherjee, S., Hudson, E., Reza, S., Thomas, M., Crosby, T., & Maughan, T. (2008). Pancreatic cancer within a UK cancer network with special emphasis on locally advanced non-metastatic pancreatic cancer. Clinical oncology (Royal College of Radiologists (Great Britain)), 20(7), 535–540. https://doi.org/10.1016/j.clon.2008.02.003 |
| Yamagishi 2010 | Wrong study design | Yamagishi, Y., Higuchi, H., Izumiya, M., Sakai, G., Iizuka, H., Nakamura, S., Adachi, M., Hozawa, S., Takaishi, H., & Hibi, T. (2010). Gemcitabine as first-line chemotherapy in elderly patients with unresectable pancreatic carcinoma. Journal of gastroenterology, 45(11), 1146–1154. https://doi.org/10.1007/s00535-010-0258-9 |
| Matsumoto 2011 | Wrong study design | Matsumoto, K., Miyake, Y., Kato, H., Kawamoto, H., Imagawa, A., Toyokawa, T., Nakatsu, M., Ando, M., Hirohata, M., & Yamamoto, K. (2011). Effect of low-dose gemcitabine on unresectable pancreatic cancer in elderly patients. Digestion, 84(3), 230–235. https://doi.org/10.1159/000330384 |
| Hiramoto 2011 | Wrong study design | Hiramoto, S., Nishida, Y., Hieda, N., Mizuguchi, A., Kakiuhi, N., Yasumura, S., Kuriyama, K., Tanabe, W., Hidaka, K., Honjo, H., Hasegawa, K., Kondou, M., Nishikawa, K., & Miyake, N. (2011). Retrospective study of chemotheraphy for unresectable advanced pancreatic cancer [Abstract]. Annals of Oncology, 22(9_suppl), ix66–ix66. https://dx.doi.org/10.1093/annonc/mdr509 |
| Hentic 2011 | Wrong study design | Hentic, O., Dreyer, C., Rebours, V., Zappa, M., Lévy, P., Raymond, E., Ruszniewski, P., & Hammel, P. (2011). Gemcitabine in elderly patients with advanced pancreatic cancer. World journal of gastroenterology, 17(30), 3497–3502. https://doi.org/10.3748/wjg.v17.i30.3497 |
| Aldoss 2011 | Wrong study design | Aldoss, I. T., Tashi, T., Gonsalves, W., Kalaiah, R. K., Fang, X., Silberstein, P., Ganti, A. K., & Subbiah, S. (2011). Role of chemotherapy in the very elderly patients with metastatic pancreatic cancer - A Veterans Affairs Cancer Registry analysis. Journal of Geriatric Oncology, 2(3), 209-214. https://doi.org/10.1016/j.jgo.2011.02.003 |
| Vijavergia 2015 | Wrong study design | Vijayvergia, N., Dotan, E., Devarajan, K., Hatahet, K., Rahman, F., Ricco, J., Lewis, B., Gupta, S., & Cohen, S. J. (2015). Patterns of care and outcomes of older versus younger patients with metastatic pancreatic cancer: A Fox Chase Cancer Center experience. Journal of geriatric oncology, 6(6), 454–461. https://doi.org/10.1016/j.jgo.2015.08.001 |
| Bednar 2016 | Wrong study design | Bednar, F., Ocuin, L. M., Steve, J., Zenati, M.S., Winters, S., Hogg, M. E., Bahary, N., Zeh, H. J., & Zureikat, A. H. (2016) FOLFIRINOX and gemcitabine/nab-paclitaxel efficacy in the treatment of locally advanced unresectable pancreatic adenocarcinoma [Abstract from 2016 ASCO Annual Meeting]. Journal of Clinical Oncology, 34(4_suppl), 399–399. http://dx.doi.org/10.1200/jco.2016.34.4_suppl.399 |
| Bednar 2016 |  | Bednar, F., Ocuin, L. M., Steve, J., Zenati, M.S., Winters, S., Hogg, M. E., Bahary, N., Zeh, H. J., Lii, H., & Zureikat, A. H. (2016) Folfirinox and gemcitabine/nab-paclitaxel demonstrate improved survival in locally advanced unresectable pancreatic adenocarcinoma [Abstract from 69th Cancer Symposium of the Society of Surgical Oncology]. Annals of Surgical Oncology, 23(1_suppl), S175–S176. |
| Chakupurakal 2017 | Wrong study design | Chakupurakal, G., Feiten, S., Burkhard, O., Reiser, M., Ehscheidt, P., & Weide, R. (2017). Successful Evidence-Based Treatment of Patients with Advanced Pancreatic Cancer in Community-Based Oncology Group Practices. Oncology research and treatment, 40(12), 784–788. https://doi.org/10.1159/000480016 |
| Henze 2018 | Wrong study design | Henze, L., Harder, P., Kragl, B., Murua Escobar, H., Grosse-Thie, C., & Junghanss, C. (2018). Approved palliative chemotherapy regimens in unresectable pancreatic cancer: About 1/3 of patients in clinical routine show differences to trial entry criteria [Abstract]. Oncology Research and Treatment, 41(4_suppl), 126–126. https://dx.doi.org/10.1159/000492737 |
| Terashima 2018 | Wrong study design | Terashima, T., Yamashita, T., Sakai, A., Ohta, H., Hinoue, Y., Toya, D., Kawai, H., Yonejima, M., Urabe, T., Noda, Y., Mizukoshi, E., & Kaneko, S. (2018). Treatment patterns and outcomes of unresectable pancreatic cancer patients in real-life practice: a region-wide analysis. Japanese journal of clinical oncology, 48(11), 966–973. https://doi.org/10.1093/jjco/hyy132 |
| Kang 2020 | Wrong study design | 1Kang, J., Lee, S. H., Choi, J. H., Paik, W. H., Ahn, D. W., Jeong, J. B., Ryu, J. K., & Kim, Y. T. (2020). Folfirinox chemotherapy prolongs stent patency in patients with malignant biliary obstruction due to unresectable pancreatic cancer. Hepatobiliary & pancreatic diseases international : HBPD INT, 19(6), 590–595. https://doi.org/10.1016/j.hbpd.2020.05.005 |
| Iede 2020 | Wrong study design | Iede, K., Yamada, T., Kato, R., Ueda, M., Tsuda, Y., Nakashima, S., Ohta, K., Matsuyama, J., Ikenaga, M., & Tominaga, S. (2020). Efficacy of S-1 in second-line chemotherapy after nab-paclitaxel plus gemcitabine for patients with advanced pancreatic cancer. Cancer reports (Hoboken, N.J.), 3(2), e1215. https://doi.org/10.1002/cnr2.1215 |
| Fukahori 2020 | Wrong study design | Fukahori, M., Okabe, Y., Shimokawa, M., Otsuka, T., Koga, F., Ueda, Y., Nakazawa, J., Komori, A., Arima, S., Makiyama, A., Taguchi, H., Honda, T., Ushijima, T., Miwa, K., Shibuki, T., Nio, K., Ide, Y., Ureshino, N., Mitsugi, K., & Shirakawa, T. (2020). Efficacy of second-line chemotherapy after standard combination chemotherapy in patients with metastatic pancreatic cancer: The results from the NAPOLEON study [Abstract from 2020 Gastrointestinal Cancers Symposium]. Journal of Clinical Oncology, 38(4_suppl), 661–661. https://dx.doi.org/10.1200/JCO.2020.38.4_suppl.661 |
| Tralongo 2020 | Wrong study design | Tralongo, A. C., Sehovic, M., Rodriquenz, M. G., Negrete Najar, J. P., Sam, C., & Extermann, M. (2020). Chemotherapy vs best supportive care in octogenarian and older stage IV pancreatic cancer patients [Abstract]. Annals of oncology, 31(4_suppl), S944–S944. https://doi.org/10.1016/j.annonc.2020.08.2019 |
| Andren-Sandberg 1983 | Wrong study design | Andrén-Sandberg, A., Holmberg, J. T., & Ihse, I. (1983). Treatment of unresectable pancreatic carcinoma with 5-fluorouracil, vincristine, and CCNU. Scandinavian journal of gastroenterology, 18(5), 609–612. https://doi.org/10.3109/00365528309181646 |
| Tsavaris 1998 | Wrong study design | Tsavaris, N., Tentas, K., Tzivras, M., Kosmas, C., Kalachanis, N., Katsikas, M., Dimitrakopoulos, A., Papastratis, G., Macheras, A., Karatzas, G., & Sechas, M. (1998). Combined epirubicin, 5-fluorouracil and folinic acid vs no treatment for patients with advanced pancreatic cancer: a prospective comparative study. Journal of chemotherapy (Florence, Italy), 10(4), 331–337. https://doi.org/10.1179/joc.1998.10.4.331 |
| Jiang 2017 | Wrong study design | Jiang, N., Qiao, G., Wang, X., Morse, M. A., Gwin, W. R., Zhou, L., Song, Y., Zhao, Y., Chen, F., Zhou, X., Huang, L., Hobeika, A., Yi, X., Xia, X., Guan, Y., Song, J., Ren, J., & Lyerly, H. K. (2017). Dendritic Cell/Cytokine-Induced Killer Cell Immunotherapy Combined with S-1 in Patients with Advanced Pancreatic Cancer: A Prospective Study. Clinical cancer research : an official journal of the American Association for Cancer Research, 23(17), 5066–5073. https://doi.org/10.1158/1078-0432.CCR-17-0492 |
| Yip 2006 | Wrong study design | Yip, D., Karapetis, C., Strickland, A., Steer, C. B., & Goldstein, D. (2006). Chemotherapy and radiotherapy for inoperable advanced pancreatic cancer. The Cochrane database of systematic reviews, (3), CD002093. https://doi.org/10.1002/14651858.CD002093.pub2 Update in: Cochrane Database Syst Rev. 2009 (4):CD002093. |
| Chin 2018 | Wrong study design | Chin, V., Nagrial, A., Sjoquist, K., O'Connor, C. A., Chantrill, L., Biankin, A. V., Scholten, R. J., & Yip, D. (2018). Chemotherapy and radiotherapy for advanced pancreatic cancer. The Cochrane database of systematic reviews, 3(3), CD011044. https://doi.org/10.1002/14651858.CD011044.pub2 |
| Nagrial 2013 |  | Nagrial, A., Chantrill, L., Chin, V., Sjoquist, K., O'Connor, C. A., & Yip, D. (2013) Pharmacologic and radiotherapeutic interventions for advanced pancreatic cancer. Cochrane Database of Systematic Reviews. Art. No.: CD010584. https://doi.org//10.1002/14651858.CD010584 |
| Betge 2018 | Wrong study design | Betge, J., Chi-Kern, J., Schulte, N., Belle, S., Gutting, T., Burgermeister, E., Jesenofsky, R., Maenz, M., Wedding, U., Ebert, M., & Haertel, N. (2018). A multicenter phase 4 geriatric assessment directed trial to evaluate gemcitabine +/− nab-paclitaxel in elderly pancreatic cancer patients (GrantPax). BMC Cancer 18, 747. https://doi.org/10.1186/s12885-018-4665-2 |
| Asahara 2013 | Wrong study design | Asahara, S., Takeda, K., Yamao, K., Maguchi, H., & Yamaue, H. (2013). Phase I/II clinical trial using HLA-A24-restricted peptide vaccine derived from KIF20A for patients with advanced pancreatic cancer. Journal of translational medicine, 11, 291. https://doi.org/10.1186/1479-5876-11-291 |
| NCT00003851 | Wrong study design | ClinicalTrials.gov Identifier: NCT00003851. Gemcitabine Compared With Pancreatic Enzyme Therapy Plus Specialized Diet (Gonzalez Regimen) in Treating Patients Who Have Stage II, Stage III, or Stage IV Pancreatic Cancer |
