# Supplementary material for: Treatment with anticancer drugs for advanced pancreatic cancer: a systematic review
Source: BMC Cancer. 2023 Aug 12;23:748. doi: 10.1186/s12885-023-11207-4 (PMC10422698; doi:10.1186/s12885-023-11207-4)
Supplement: Supplementary file 3 — Additional file 3. Characteristics of included studies. [file 12885_2023_11207_MOESM3_ESM.docx]

**Characteristics of included studies**

| **Study ID** | **Eligibility criteria** | **Intervention** | | **Comparison** | |
| --- | --- | --- | --- | --- | --- |
|  |  | **Name** | **Description** | **Name** | **Description** |
| Chemotherapy | | | | | |
| Frey, 1981 | - Histologically confirmed  - Unresectable PC  - Condition permitted drug therapy to begin between 10 and 60 days postoperatively. | 5-FU + CCNU + Celiotomy/Biliary bypass | 5-FU 9 mg/kg i.v. on 5 consecutive days. CCNU 70 mg/m2 orally on the first day of cycle. The cycle was repeated every 6 weeks. | Palliative Surgery | No drug therapy. |
| Glimelius, 1996 | Inclusion  - Histologically confirmed - Non curable Pancreatic adenocarcinoma  Exclusion  - Serum creatinine level > 125 mmol/1, serum bilirubin level > 60 mmol/1 - Other primary tumours - KPS <50% - Previous chemotherapy | 5-FU + Leucovorin ± Etoposide + BSC | >60 years with KPS 70 or less: 5-FU 500 mg/m2 bolus i.v. and leucovorin 60 mg/m2 bolus i.v. 40 minutes later on 2 consecutive days every second week. Otherwise: 5-FU 500 mg/m2 bolus i.v. followed by etoposide 120 mg/m2 i.v. infusion during 40 minutes and leucovorin 60 mg/m2 bolus i.v. on 3 consecutive days every third week | BSC | Included psychosocial support and attempts to relieve any symptoms (analgesics, antiemetic drugs, nutritional support, corticosteroids, palliative radiotherapy, surgery and so on). Chemotherapy was allowed if the supportive measures did not result in palliation. |
| Huguier, 2001 | Inclusion  - Histologically confirmed - Unresectable Pancreatic adenocarcinoma  Exclusion  - Neuroendocrince tumors, mucinous carcinoma or other malignant pancreatic tumors, cholangiocarcinomas, and ampullary carcinomas.  - Active heart disease, abnormal creatinin clearance, poor general condition  - WHO PS >2  - Other contraindications to chemotherapy. | 5-FU + Cisplatin + Leucovorin + SC | 5 day course of leucovorin 200 mg/m2/day bolus i.v. followed by infusion of 5-FU 375mg/m2/day in 1L saline over 2 hours, followed by infusion of cisplatin 15mg/m2/day in 250 mL of saline over 1 hour. After CDDP administration, 1L of saline was infused over 2 hours. Cycles were repeated every 21 days. In the absence of gastrointestinal, renal or hematologic toxicities, the daily dose of 5-FU was increased by 25mg/m2/day at each subsequent cycle (the max daily dose was 550mg/m2/day) | SC | Not described. Includes palliative bypass, splanchinectomy, palliative resection. |
| Mallinson, 1980 | Inclusion  - 35 to 75 years - Unresectable PC  Exclusion  - Unable to visit the outpatient clinic regularly - Impaired renal function, unrelievable gastrointestinal obstruction, neutropenia or thrombocytopenia  - Had already received radiotherapy or cancer chemotherapy | 5-FU + Methrotexate + Vincristine + Cyclophosphamide | Initiation course of 5-FU all days 500 mg or 7.5 mg/kg if outside 60-80kg, methrotexate days 1 and 4 20 mg or 0.3 mg/kg if outside of 60-80kg, vincristine days 2 and 5 1 mg or 0.02 mg/kg if outside of 60-80 kg, cyclophosphamide on days 1and 5 200 mg or 4.5 mg/kg if outside 60-80kg, given in hospital over 5 days. All drugs were given i.v. as separate bolus injections followed by 5 ml of normal saline. Follow-up treatment (4 weeks after the end of the initiation course) consisted of intravenous 5-FU, 10 mg/kg body weight, and mitomycin, 100 ug/kg body weight by i.v. bolus injection, daily on five consecutive days and repeated every six weeks until death or for two years. | SC | Analgesics and other supportive treatment. |
| The Gastrointestinal Tumor Study Group, 1979 | Inclusion  - Histologically confirmed  - Unresectable locally advanced adenocarcinoma of the pancreas  - Apparently confined to the pancreas, regional lymph nodes, regional peritoneum or peripancreatic organs in continuity, if the entire area of malignant disease could be encompassed within a 400 cm2 area.  Exclusion  - Islet cell carcinoma or cystadenocarcinoma of the pancreas - Coexistent malignant disease  - Prior chemotherapy or radiation therapy | Intervention 1: 5-FU + 4000R Intervention 2: 5-FU + 6000R | 5FU 500 mg/M2/day bolus i.v. the first three days of RT. Following completion of RT, 5FU was adminitered at a dose of 500 mg/M2 i.v. weekly for 4 weeks | Palliative Radiotherapy | 6000R + No additional therapy |
| Xinopoulos, 2008 | Inclusion  - 25 to 75 years  - Histologically or cytologically confirmed - Locally advanced PC - No prior anticancer therapy - No indication for radiotherapy  - KPS > 50% - Life expectancy > 3 months - Absence of duodenal obstruction - No previous biliary stent placement, gastrectomy, choledochoduodenostomy, choledochojejunostomy or hepaticojejunostomy.  - Adequate pulmonary and renal function, satisfactory liver biochemistry after stenting, INR ≤ 1.4, adequate bone marrow reserve  - No evidence of viral, autoimmune and hereditary liver disease.  Exclusion  - Concomitant malignancy, central nervous system metastatic disease, severe heart disease, severe neurological impairment or mental disorder, BM difficult to control, pulmonary fibrosis or interstitial pneumonia, marked peripheral edema, marked pericardial or pleural effusion, active infection - Pregnancy and lactation - Ineffective contraception for females of childbearing age  - Severe drug hypersensitivity | Gemcitabine + Palliative surgery | First course during surgery, then administered as an i.v. 30 min infusion of 1000 mg/m2 per week for 3 consecutive weeks followed by 1-week rest of each 28-day cycle. | Palliative Surgery | No further treatment. |
| Ciuleanu, 2009 | Inclusion  - ≥18 years. - Metastatic pancreatic adenocarcinoma - Distant metastasis - At least one target or non-target lesion by RECIST.  - Progressed during or after treatment with gemcitabine - Recovered from reversible toxicities of prior therapy - Adequate organ reserve including haematopoietic, hepatic and renal function - KPS ≥ 70%  Exclusion  - More than 1 prior systemic therapy regimen for advanced disease. | Glufosfamide + BSC | 4500 mg/M2 i.v. over 6 hours on day 1 of every 3-week cycle | BSC | Analgesics, antibiotics, transfusions, therapeutic haematopoietic colony-stimulating factors, erythropoietin and other appropriate supportive measures including concomitant medications that do not have anti-tumour effects. Megestrol acetate for appetite stimulation was permitted. |
| Pelzer, 2011 | Inclusion  - ≥18 years. - Histologically confirmed - Advanced Pancreatic adenocarcinoma - Progressed during first line gemcitabine  - KPS > 60% - Measurable reference lesion  - Adequate laboratory values for haematology, renal and hepatic function  Exclusion  - Any severe concurrent medical condition interfering with the planned therapy, serious cardiac disease, sensory/motor neuropathy >grade 2, uncontrolled pain  - Previous or active malignancies of other origin  - Pregnant or breastfeeding women  - Prior radiotherapy with or without chemosensitization  - Recurrence while receiving adjuvant gemcitabine post curative surgery. | OFF (5-FU + Oxaliplatin + Leucovorin) | 6 weeks cycle of leucovorin 0.2 g/m2, 0.5h, i.v. followed by 5-FU 2 g/m2, 24-h, i.v. administered on days 1, 8, 15 and 22. Oxaliplatin 0.85 g/m2, 2–4 h, i.v. prior to Leucovorin/5-FU on days 8 and 22. After a 3 weeks rest (days 23 to 42) the next cycle was started (day 43 = day 1 of next cycle). | BSC | Adequate pain management, therapy of infection, biliary-stent intervention if needed, social supply and on demand psychooncologically intervention and nutrition consultation/intervention. Patients in the BSC arm regularly were seen in the outpatient department at least every 14 d. They were visited not so often like patients in the treatment group, but if needed the visit number was equal or even higher. |
| Palmer, 1994 | Inclusion  - Unresectable advanced PC.  - Diagnosis: radiographic findings at ultrasonography, CT and ERCP. Percutaneous fine-needle aspiration, biopsy of primary or sccondary tumour or examination of tissue obtained at laparotomy. - Staged using ultrasonography, CT and laparotomy  Exclusion  - Previous malignancy (excluding basal or squamous carcinoma of the skin)  - Significant renal or cardiovascular disease, thrombocytopenia or leucopenia  - Gross psychiatric disease  - WHO PS > 3. | 5-FU + Adriamycin + Mitomycin | 8-week cycle. 5-FU 600mg/m2 i.v was given on days 1 and 29, and oral 5-FU on days 8 and 36; Adriamycin 30mg/m2 bolus i.v. on days 1 and 29; mitomycin 10mg/m2 i.v. was given on days 1 and 29. | SC | Not described. |
| Shinchi, 2002 | Inclusion  - Histologically or cytologically confirmed - Unresectable locally advanced PC - Without distant metastases - Determined by CT, magnetic resonance cholangiography, ultrasonography, or angiography.  - KPS ≥ 60% - Adequate biliary drainage, normal excretion from both kidneys | 5-FU + EBRT | EBRT delivered with 10-MV photons using a four-field technique in fractions of 1.8–2.0 Gy/day, 5 days per week. An average total dose of 50.8 Gy was administered (range: 25.2–60 Gy) over 3 to 7 weeks. 5-FU 200 mg/m2/day i.v., 24 h/day, for the duration of their radiation therapy. One week after the completion, 5-FU 500 mg/m2 bolus i.v. was given weekly. | BSC | For symptoms such as pain, anorexia, weight loss, and so on. Also includes palliative biliary bypass procedures. |
| Takada, 1998 | Inclusion  - <75 years - Histologically confirmed - Unresectable PC - Japanese Cancer Therapy Society Chemotherapy Efficacy Evaluation Criteria PS 0-3 - No simultaneous, heterochronic multiple or recurrent carcinoma. - No severe complications.  - WBC >4000 mm3, PLT >100000/mm3, GPT <100 U and urinary protein (-) in their laboratory findings at the start of treatment. | Modified FAM (5-FU + Doxorubicin + Mitomycin) + Palliative surgery | 5-FU 200mg/m2, Doxorubicin 15mg/m 2, and Mitomycin 5mg/m2 by i.v. started on the same day of their palliative surgery. Therapy was given once per week for 4 weeks, followed by 1 week with no therapy. The therapy was repeated for two complete courses. | Palliative Surgery | No drug therapy. |
| Immunotherapy | | | | | |
| Oortgiesen, 2010 | Inclusion  - Adult patients - Histologically or cytologically confirmed - Unresectable advanced PC - Stage II, III or IV - Life expectancy > 2 months | PAS Vaccination | Intramuscularly on weeks 0, 1, 3, and 24. | Placebo | NR |
| Gilliam, 2012 | Inclusion  - Histologically confirmed - Advanced PC unsuitable or unwilling to receive chemotherapy  - KPS ≥ 60% - Life expectancy > 2 months  Exclusion  - Any other malignant disease within the previous 5 years - Any prior anticancer therapy  - Known immunodeficiency or any other condition that would worsen during the study period, reduce subject compliance, or confound interpretation of data.  - Patients with hematological derangement that might potentially reduce immunologic response - Pregnant, lactating, or of childbearing potential - Subjects who had participated in other studies 3 months preceding enrolment. | G17DT: Antigastrin immunogen | 250 mg at weeks 0, 1, 3, 24, and 52 via an intramuscular injection to the thigh. An additional dose of G17DT was offered at 18 months. | Placebo | Same 0.2-mL volume of vehicle at weeks 0, 1, 3, 24, and 52 via an intramuscular injection to the thigh. |
| Targeted/biological therapy | | | | | |
| Propper, 2014 | Inclusion  - ≥18 years. - Histologically or cytologically confirmed - Measurable disease (by RECIST) and accesible for biopsy - Unresectable locally advanced or metastatic PC - Failed on prior chemotherapy or were deemed unsuitable for first-line chemotherapy. - ECOG PS 0–2 - Life expectancy of ≥6 weeks - Adequate hematologic, renal and hepatic function  Exclusion  - Resectable PC - Other malignancies in the last 5 years (except cervical, basal or squamous-cell skin cancer) - Major surgery <2 weeks before randomization - Spinal cord compression or central nervous system metastases | Erlotinib | 150 mg/day | Placebo | Matching to erlotinib 150 mg tablet. Orally once daily. |

RCT: randomized clinical trial; PC: pancreatic cancer; PAC: pancreatic adenocarcinoma; PS: performace status; KPS: Karnosfky PS; CT: chemotherapy; RT: radiotherapy; BSC: best supportive care; SC: supportive care; CCNU: Lomustine, 1 - [2-chlorethyl - 3-cyclohexyl - 1 nitrosourea; 5FU: fluorouracil; EBRT: external beam radiotherapy; PAS: polyclonal antibody stimulator; ECOG: Eastern Cooperative Oncology Group; ERCP: Endoscopic retrograde cholangiopancreatography; NR: Not reported
