# Supplementary material for: Treatment with anticancer drugs for advanced pancreatic cancer: a systematic review
Source: BMC Cancer. 2023 Aug 12;23:748. doi: 10.1186/s12885-023-11207-4 (PMC10422698; doi:10.1186/s12885-023-11207-4)
Supplement: Supplementary file 4 — Additional file 4. Judgements for each risk of bias domain for included studies. [file 12885_2023_11207_MOESM4_ESM.docx]

**Judgements for each risk of bias domain for included studies**


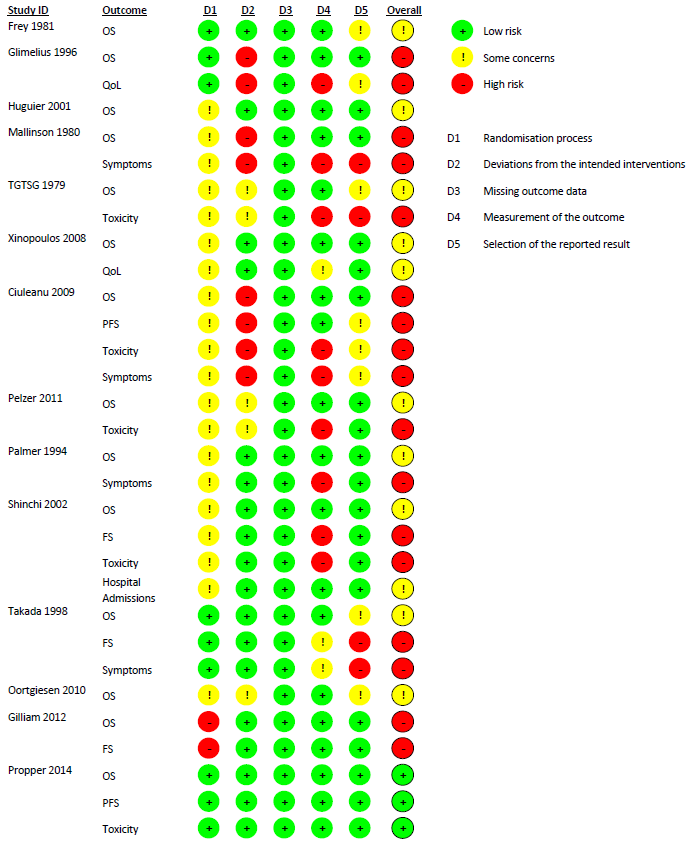


TGTSG: The Gastrointestinal Tumour Study Group
