# Supplementary material for: Treatment with anticancer drugs for advanced pancreatic cancer: a systematic review
Source: BMC Cancer. 2023 Aug 12;23:748. doi: 10.1186/s12885-023-11207-4 (PMC10422698; doi:10.1186/s12885-023-11207-4)
Supplement: Supplementary file 5 — Additional file 5. Results for chemotherapy and targeted/biological therapy versus no-TAD per outcome. [file 12885_2023_11207_MOESM5_ESM.docx]

**Results for chemotherapy and targeted/biological therapy versus no-TAD per outcome.**

1. Chemotherapy versus no-TAD. Outcome: Overall survival (OS)

**Median OS**

| Study ID | Chemotherapy | Control |
| --- | --- | --- |
| Frey, 1981 (1) | 3 m | 3.9 m |
| Glimelius, 1996 (2) | 6 m | 2.5 m |
| Huguier, 2001 (3) | 8.6 m* | 7.0 m* |
| Mallinson, 1980 (4) | 44 w | 9 w |
| TGTSG, 1979 (5) | 36 w (Int 1)  40 w (Int 2) | 20 w |
| Xinopoulos, 2008 (6) | 21 w | 22 w |
| Ciuleanu, 2009 (7) | 105 d | 84 d |
| Pelzer, 2011 (8) | 21 w | 10 w |
| Palmer, 1994 (9) | 33 w | 15 w |
| Shinchi, 2002 (10) | 13.2 m | 6.4 m |
| Takada, 1998  (11) | NR | NR |

TGTSG: The Gastrointestinal Tumor Study Group; d: days; w: weeks; m: months; NR: not reported; Int: intervention. *: mean

**OS (months)**


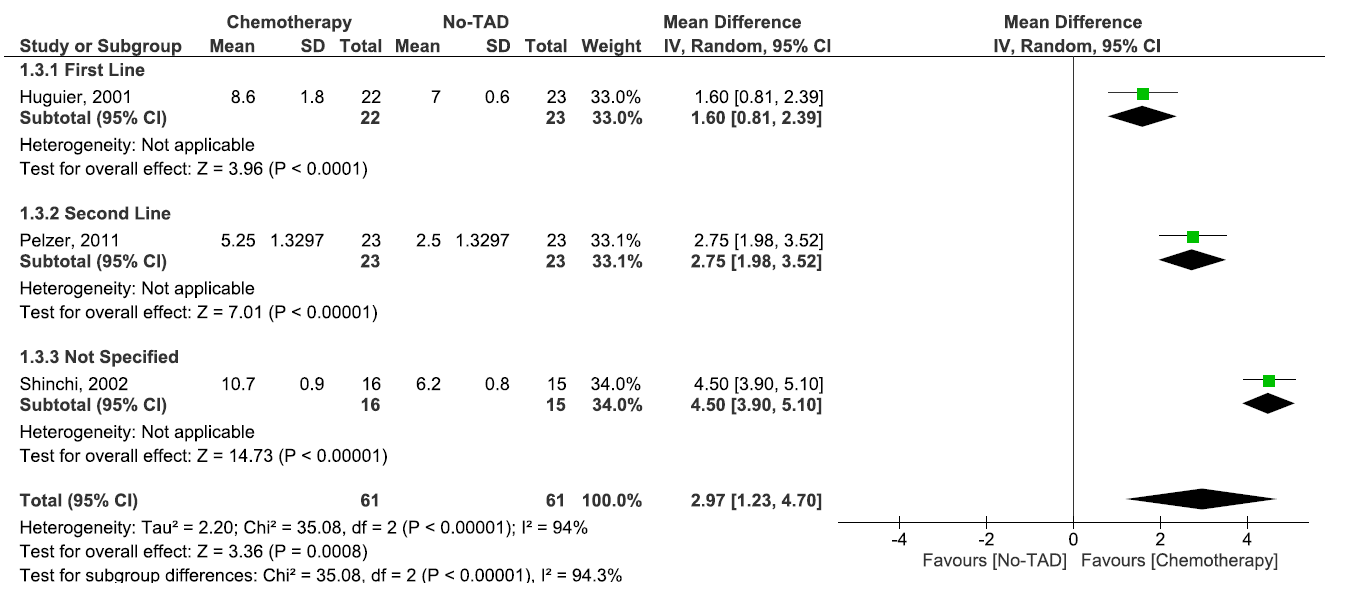


**OS (time to event)**


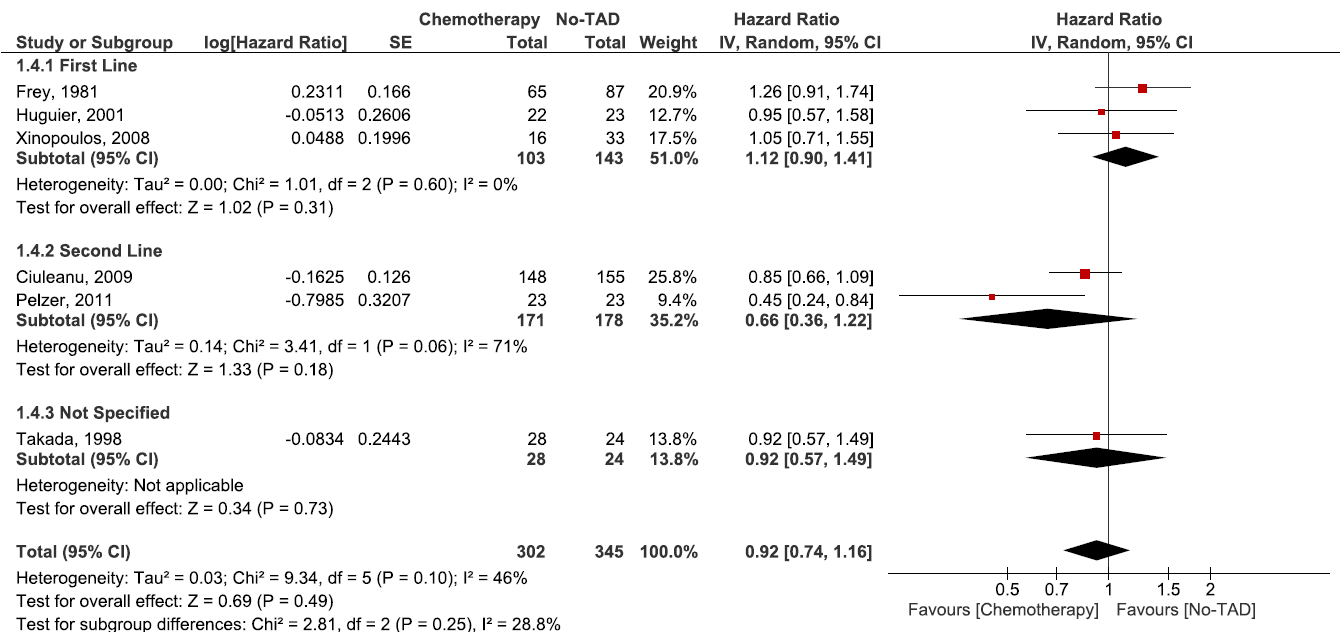


2. Chemotherapy versus No-TAD. Outcome: Mortality

**3-month mortality**


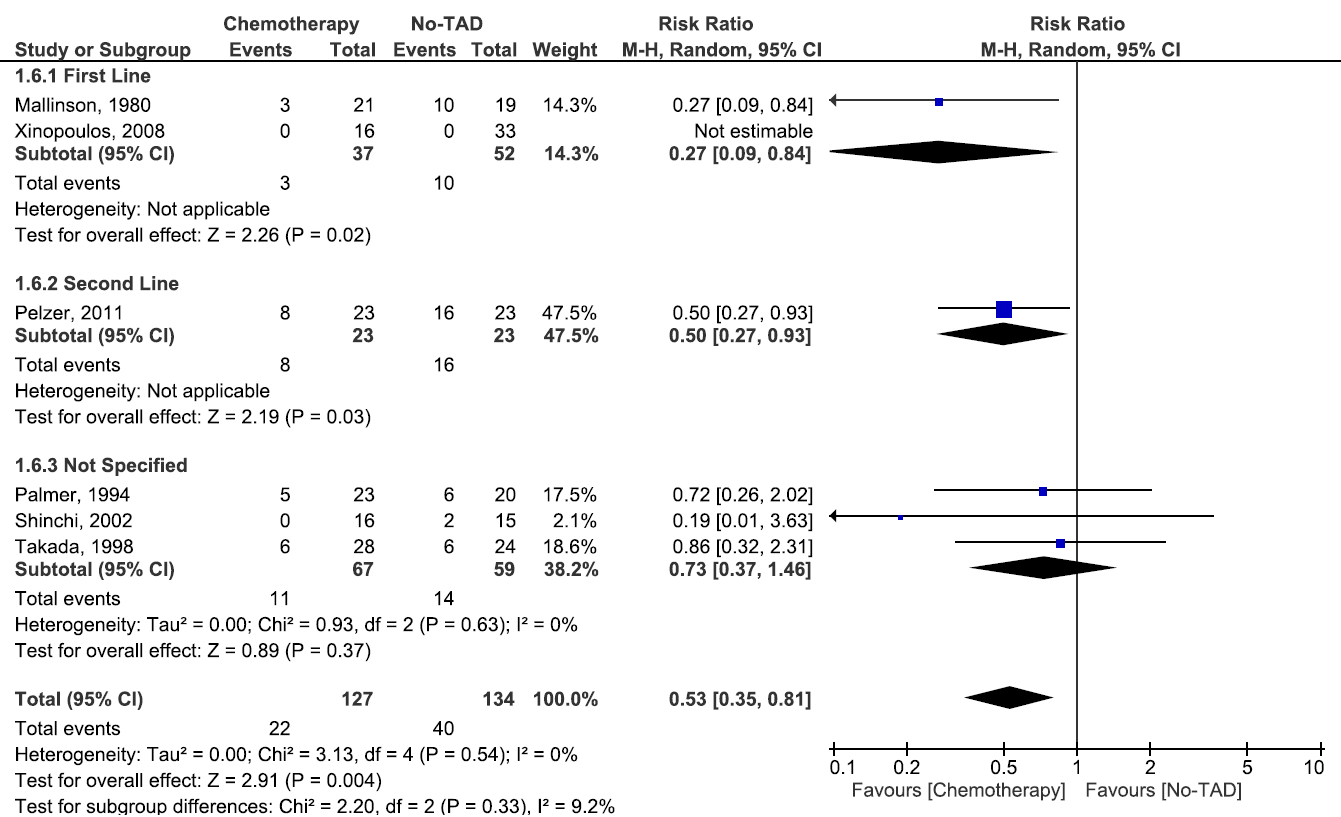


**6-month mortality**


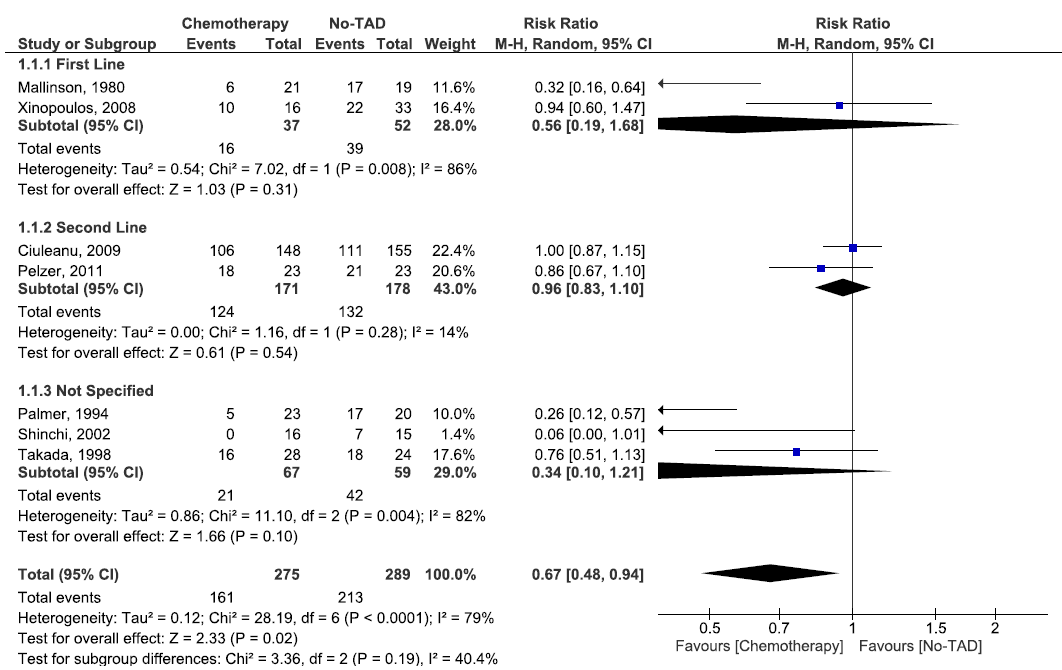


**
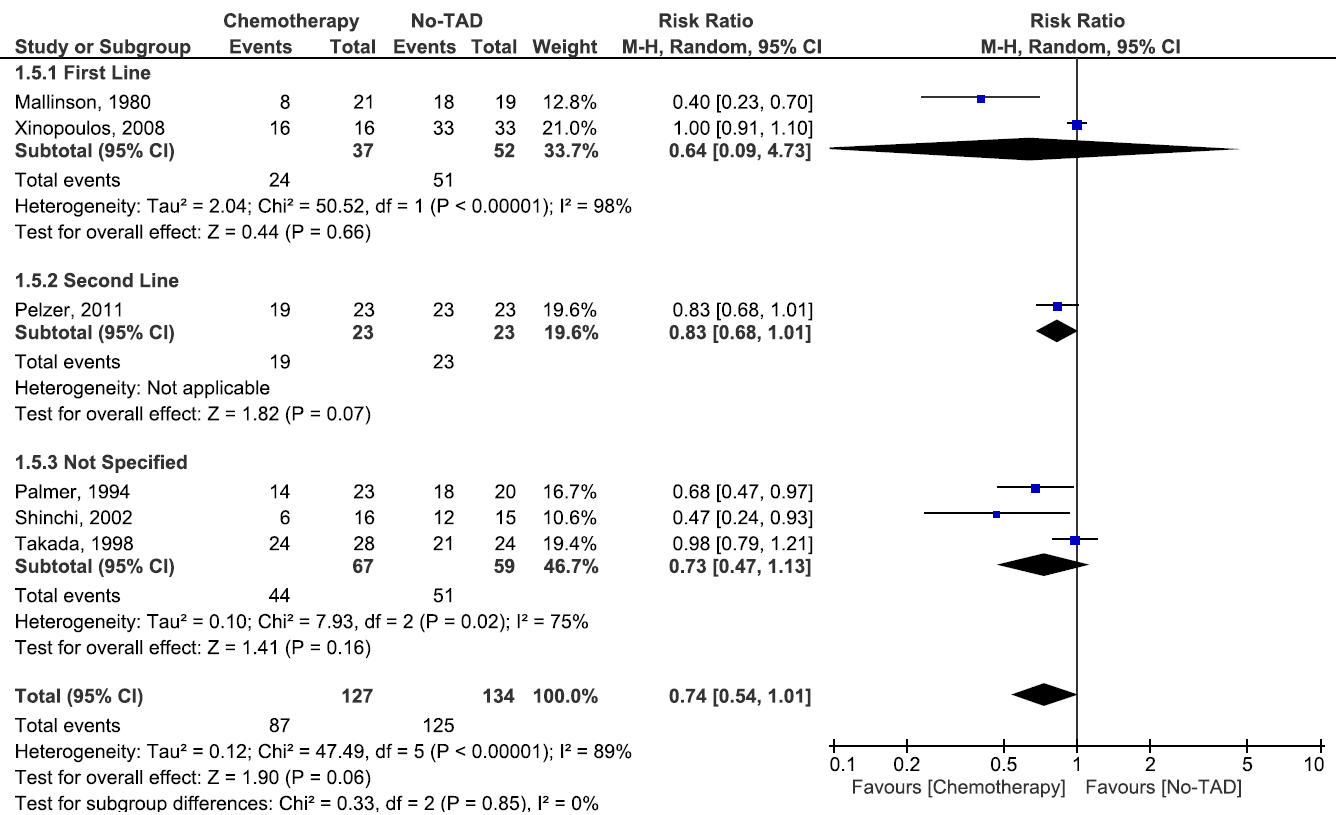
9-month mortality**

**12-month mortality**


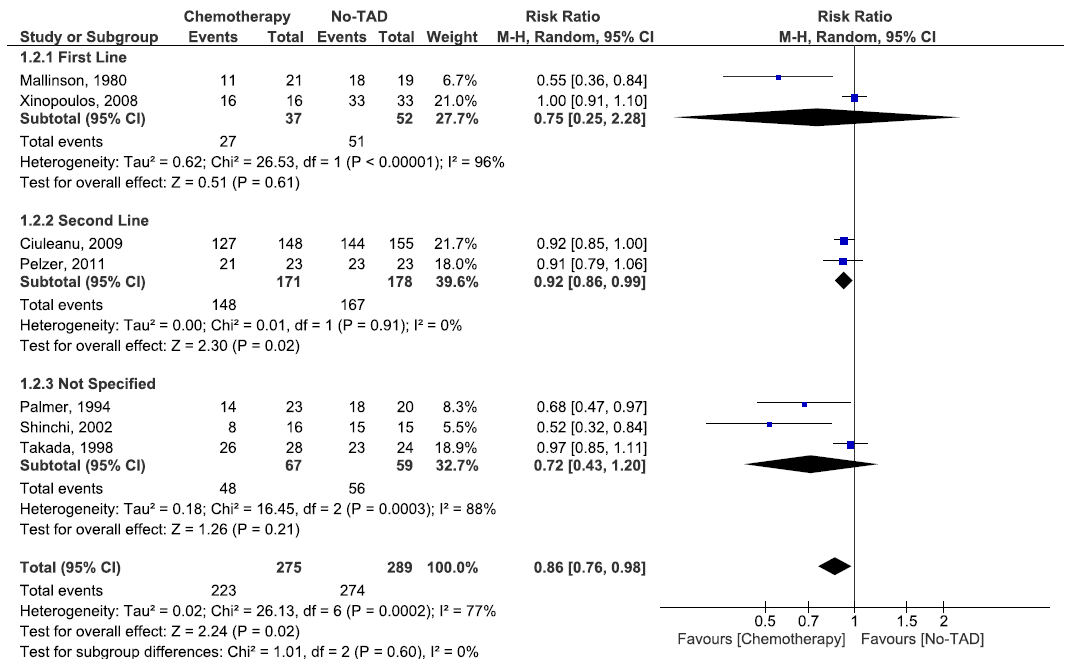


3. Chemotherapy/ Targeted/biological therapy versus No-TAD. Outcome: Adverse Events

| **Study ID** | **Toxicity** | | | **Intervention** | | **Control** | |
| --- | --- | --- | --- | --- | --- | --- | --- |
|  | **Scale** | **Grade** | **Description** | **n** | **N** | **n** | **N** |
| Chemotherapy | | | | | | | |
| Shinchi, 2002 | WHO’s recommendations for grading of acute and subacute effects (1979) | 3 | Anorexia and Nausea | 1 | 16 | 0 | 15 |
| Pelzer, 2011 | National Cancer Institute’s Common Toxicity Criteria (CTCAE) | 3 | Diarrhoea | 2 | 23 | 0 | 23 |
|  |  |  | Nausea/emesis | 1 | 23 | 0 | 23 |
|  |  |  | Thrombocytopenia | 0 | 23 | 1 | 23 |
|  |  |  | Anaemia | 0 | 23 | 1 | 23 |
|  |  |  | Paraesthesia | 0 | 23 | 1 | 23 |
|  |  |  | Leukopenia | 0 | 23 | 0 | 23 |
|  |  | 4 | Leukopenia | 0 | 23 | 0 | 23 |
|  |  |  | Nausea/emesis | 0 | 23 | 0 | 23 |
|  |  |  | Thrombocytopenia | 0 | 23 | 0 | 23 |
|  |  |  | Diarrhoea | 0 | 23 | 0 | 23 |
|  |  |  | Anaemia | 0 | 23 | 0 | 23 |
|  |  |  | Paraesthesia | 0 | 23 | 0 | 23 |
| The Gastrointestinal Tumour Study Group, 1979 | NR | 3 or 4 | Nausea and vomiting | 3/89 | | | |
|  |  | 5 | Leukopenia and sepsis | 1* | 29* | 0 | 28 |
|  |  |  |  | 1** | 32** |  |  |
| Ciuleanu, 2009 | National Cancer Institute’s Common Toxicity Criteria (CTCAE v3.0) | 3, 4 and 5 | Any adverse event | 63 | 141 | 44 | 145 |
|  |  |  | Asthenia/fatigue | 12 | 141 | 11 | 145 |
|  |  |  | Abdominal pain | 11 | 141 | 13 | 145 |
|  |  |  | Anaemia | 7 | 141 | 3 | 145 |
|  |  |  | Vomiting | 7 | 141 | 2 | 145 |
|  |  |  | Nausea | 6 | 141 | 2 | 145 |
|  |  |  | Deep vein thrombosis | 5 | 141 | 1 | 145 |
|  |  |  | Renal Failure | 5 | 141 | 0 | 145 |
|  |  |  | Hyperbilirubinemia | 4 | 141 | 2 | 145 |
|  |  |  | Leukopenia | 4 | 141 | 0 | 145 |
|  |  |  | Anorexia | 3 | 141 | 2 | 145 |
|  |  |  | Hyperglycaemia | 3 | 141 | 1 | 145 |
|  |  |  | Hypokalaemia | 3 | 141 | 0 | 145 |
| Xinopoulos, 2008 | NR | 1, 2 and 3 | Thrombocytopenia | 10 | 16 | NR | |
|  |  | 2 and 3 | Anaemia | 5 | 16 |  |  |
| Huguier, 2001 | WHO toxicity | 3 and 4 | Any | 0 | 22 | NR | |
| Palmer, 1994 | WHO toxicity | 3 | Alopecia | 11 | 22 | NR | |
|  |  |  | Nausea and vomiting | 6 | 22 |  |  |
|  |  |  | Oral ulceration | 2 | 22 |  |  |
|  |  |  | Leukopenia | 1 | 22 |  |  |
|  |  | 4 | Leukopenia | 2 | 22 |  |  |
|  |  |  | Oral ulceration | 1 | 22 |  |  |
|  |  |  | Alopecia | 0 | 22 |  |  |
|  |  |  | Nausea and vomiting | 0 | 22 |  |  |
| Targeted/biological Therapy | | | | | | | |
| Propper, 2014 | National Cancer Institute’s Common Toxicity Criteria (CTCAE v3.0) | 3 | Any | 38 | 104 | 34 | 103 |
|  |  | 4 | Any | 2 | 104 | 4 | 103 |
|  |  | 5 | Any | 5 | 104 | 2 | 103 |
|  |  | Leading to death | | 5 | 104 | 2 | 103 |
|  |  | Patients with ≥1 serious AE | | 21 | 104 | 11 | 103 |
|  |  | Patients with ≥1 treatment-related serious AE | | 11 | 104 | 2 | 103 |

AE: adverse event; NR: not reported; *Intervention group 1. **Intervention group 2

4. Chemotherapy versus No-SOT. Outcome: Symptoms related to the disease

| **Study ID** | **Follow-up** | **Scale** | **Description** | **Intervention** | | **Control** | |
| --- | --- | --- | --- | --- | --- | --- | --- |
|  |  |  |  | **n** | **N** | **n** | **N** |
| Ciuleanu, 2009 | 2 consecutive cycles | Pain intensity in the previous 24 h using a 100-mm visual analogue scale (VAS) performed on day 1 of every cycle. | No increase in VAS pain scores | 72%^a^ | | 44%^a^ | |
| Mallinson, 1980 | NR | Participants ever experiencing symptoms | Pain | 19 | 21 | 11 | 16 |
|  |  |  | Nausea | 17 | 21 | 7 | 16 |
|  |  |  | Vomiting | 15 | 21 | 7 | 16 |
|  |  |  | Diarrhoea | 11 | 21 | 5 | 16 |
| Palmer, 1994 | Baseline | Hospital Anxiety and Depression (HAD) questionnaire^b^ | case level depressionb | 2 | 18 | 6 | 13 |
|  |  |  | case level anxiety | 8 | 18 | 6 | 13 |
|  | 2 months |  | case level depression | 2 | 15 | 4 | 6 |
|  |  |  | case level anxiety | 4 | 15 | 1 | 6 |
| Takada, 1998 | NR | Any improvement in symptoms with at least 2 kg body weight gain | | 4 | 28 | 1 | 24 |

^a^ number of participants not reported by study authors, analysis only included subjects on study for at least 3 cycles

^b^ case level defined by study authors as clinically significant symptoms.
