# Supplementary material for: Treatment with anticancer drugs for advanced pancreatic cancer: a systematic review
Source: BMC Cancer. 2023 Aug 12;23:748. doi: 10.1186/s12885-023-11207-4 (PMC10422698; doi:10.1186/s12885-023-11207-4)
Supplement: Supplementary file 6 — Additional file 6. Summary of findings for chemotherapy compared to no-TAD for advanced pancreatic cancer. [file 12885_2023_11207_MOESM6_ESM.docx]

Summary of findings for chemotherapy compared to no-TAD for advanced pancreatic cancer.

| **Chemotherapy compared to no-TAD for advanced pancreatic cancer** | | | | | |
| --- | --- | --- | --- | --- | --- |
| **Outcomes** | **№ of participants (studies) Follow-up** | **Certainty of the evidence (GRADE)** | **Relative effect (95% CI)** | **Anticipated absolute effects** | |
|  |  |  |  | **Risk with no-TAD** | **Risk difference with chemotherapy** |
| Mortality 6 months | 564 (7 RCTs) ^1,2,3,4,5,6,7^ 6 months | ⨁◯◯◯ Very low ^a,b,c^ | RR 0.67 (0.48 to 0.94) | 768 per 1,000 | 253 fewer per 1,000 (399 fewer to 46 fewer) |
| Mortality 12 months | 564 (7 RCTs) ^1,2,3,4,5,6,7^ 12 months | ⨁◯◯◯ Very low ^a,c,d^ | RR 0.86 (0.76 to 0.98) | 962 per 1,000 | 135 fewer per 1,000 (231 fewer to 19 fewer) |
| *The risk in the intervention group (and its 95% confidence interval) is based on the assumed risk in the comparison group and the relative effect of the intervention (and its 95% CI).  CI: confidence interval; HR: hazard Ratio; MD: mean difference; RR: risk ratio | | | | | |
| **GRADE Working Group grades of evidence High certainty: we are very confident that the true effect lies close to that of the estimate of the effect. Moderate certainty: we are moderately confident in the effect estimate: the true effect is likely to be close to the estimate of the effect, but there is a possibility that it is substantially different. Low certainty: our confidence in the effect estimate is limited: the true effect may be substantially different from the estimate of the effect. Very low certainty: we have very little confidence in the effect estimate: the true effect is likely to be substantially different from the estimate of effect.** | | | | | |
| Explanations a. We downgraded two levels due to some concerns of risk of bias of most included studies, mainly due to lack of information about allocation concealment. Additionally, we judged two studies (Ciuleanu et al, and Mallinson et al.) to have a high risk of bias tue to deviations from interventions, and through sensitivity analysis we found that their exclusion from metanalysis impacted precision of the estimates, crossing the line of no effect. b. We downgraded one level due inconsistency. There was considerable statistical heterogeneity (I2: 79%) but most studies tended to favour the chemotherapy arm. c. We downgraded one level due to imprecision. We considered for small, moderate, and large effects as 5, 10 and 15% reduction in mortality, respectively. The estimate's confidence interval crosses all these thresholds. d. We downgraded one level due inconsistency. There was considerable statistical heterogeneity (I2: 77%) but most studies tended to favour the chemotherapy arm. | | | | | |
| References 1.Pelzer U, Schwaner I,Stieler J,Adler M,Seraphin J,Dörken B,Riess H,Oettle H. Best supportive care (BSC) versus oxaliplatin,folinic acid and 5-fluorouracil (OFF) plus BSC in patients for second-line advanced pancreatic cancer: a phase III-study from the German CONKO-study group. Eur J Cancer. 2011 Jul, 47(11):1676-81, . . 2.Shinchi H, Takao S,Noma H,Matsuo Y,Mataki Y,Mori S,Aikou T. Length and quality of survival after external-beam radiotherapy with concurrent continuous 5-fluorouracil infusion for locally unresectable pancreatic cancer. Int J Radiat Oncol Biol Phys. 2002 May 1, 53(1):146-50., . . 3.Xinopoulos D, Dimitroulopoulos D,Karanikas I,Fotopoulou A,Oikonomou N,Korkolis D,Kouroumalis E,Antsaklis G,Vassilopoulos P,Paraskevas E. Gemcitabine as palliative treatment in patients with unresectable pancreatic cancer previously treated with placement of a covered metal stent. A randomized controlled trial. J BUON. 2008 Jul-Sep, 13(3):341-7., . . 4.Ciuleanu TE, Pavlovsky AV,Bodoky G,Garin AM,Langmuir VK,Kroll S,Tidmarsh GT. A randomised Phase III trial of glufosfamide compared with best supportive care in metastatic pancreatic adenocarcinoma previously treated with gemcitabine. Eur J Cancer. 2009 Jun, 45(9):1589-96., . . 5.Takada T, Nimura Y,Katoh H,Nagakawa T,Nakayama T,Matsushiro T,Amano H,Wada K. Prospective randomized trial of 5-fluorouracil,doxorubicin,and mitomycin C for non-resectable pancreatic and biliary carcinoma: multicenter randomized trial. Hepatogastroenterology. 1998 Nov-Dec, 45(24):2020-6., . . 6.Mallinson CN, Rake MO,Cocking JB,Fox CA,Cwynarski MT,Diffey BL,Jackson GA,Hanley J,Wass VJ. Chemotherapy in pancreatic cancer: results of a controlled,prospective,randomised,multicentre trial. Br Med J. 1980 Dec 13, 281(6255):1589-91., . . 7.Palmer KR, Kerr M,Knowles G,Cull A,Carter DC,Leonard RC. Chemotherapy prolongs survival in inoperable pancreatic carcinoma. Br J Surg. 1994 Jun, 81(6):882-5., . . | | | | | |
